# Establishment and validation of a prognostic risk model based on ADME-related genes in breast cancer

**DOI:** 10.3389/fonc.2025.1568379

**Published:** 2025-11-07

**Authors:** Yang Yang, Lei Yan, Yang Feng, Yuling Liu, Guangmin Shi, Jiqing Hao

**Affiliations:** 1 Department of Oncology Ward 2, Suzhou Hospital of Anhui Medical University, Suzhou, Anhui, China; 2 Department of Oncology, The First Affiliated Hospital of Anhui Medical University, Hefei, Anhui, China; 3 Department of Pathology, Suzhou Hospital of Anhui Medical University, Suzhou, Anhui, China; 4 Central Laboratory, Suzhou Hospital of Anhui Medical University, Suzhou, Anhui, China

**Keywords:** breast cancer, absorption, distribution, metabolism, excretion, prognostic genes

## Abstract

**Background:**

The processes of absorption, distribution, metabolic action, and elimination (ADME) affect the advancement of cancer and the development of resistance to therapies. This study examined ADME-related genes in breast cancer (BRCA) mechanisms and their associations with BRCA.

**Methods:**

BRCA datasets were analyzed to identify genes with differential expression in BRCA compared to normal tissues, focusing on ADME-related genes (ADME-RGs). Stepwise regression analyses identified prognostic genes, which were used to develop a risk assessment model. BRCA patients were scored and classified into risk categories, with survival outcomes compared across groups. A predictive model incorporating key prognostic indicators estimated patient survival rates. Mechanisms were explored through enrichment analysis, immune profiling, and drug sensitivity testing. Quantitative reverse transcription polymerase chain reaction (qRT-PCR) and western blot (WB) methodologies were employed to determine the transcription and translation levels of the six genes, with immunohistochemistry (IHC) used to validate the variations in their expression profiles.

**Results:**

Findings indicated that six predictive genes were pinpointed which established a risk stratification model, categorizing individuals into groups with either high or low risk, whereas those in the low-risk category demonstrated improved survival outcomes. A nomogram was created for precise prediction. Analysis of enrichment pinpointed processes, including metabolism of arachidonic and fatty acids, regulation of cellular division, proteasomal activity, and breakdown of tyrosine. Immune infiltration analysis showed distinct profiles for seven cell types between risk groups. Drug sensitivity analysis revealed GW.441756, imatinib, and WH.4.023 were more effective in the low-risk group, with varying sensitivities to other drugs in the high-risk group. The qRT-PCR, WB, and IHC results matched the bioinformatics analysis, showing upregulated ATP7B expression in BRCA, indicating the high prognostic potential of the identified genes.

**Conclusions:**

ADME-related prognostic genes (GSTM2, ADHFE1, ALDH2, NOS1, ATP7B, and ALDH3A1) are implicated in BRCA pathogenesis, suggesting new therapeutic strategies for BRCA treatment.

## Graphical Abstract

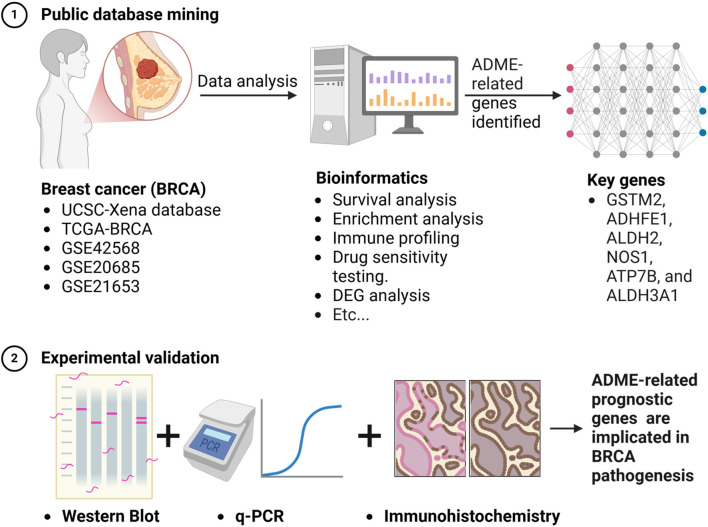

## 1 Introduction

Currently in 2022, the most common cancer affecting women worldwide was breast cancer (BRCA), defined by the presence of cancerous growths that develop from the epithelial cells of breast tissue (1). Although there have been notable improvements in treatment options, such as radiation therapy, drug-based cancer treatments, hormone therapy, and precision medicine approaches, many patients continue to experience poor outcomes due to distant metastases, with low overall survival rates (2, 3). BRCA remains a major cause of cancer deaths (1), underscoring the pressing need to identify prognostic genes that can facilitate outcome prediction and inform personalized treatment strategies.

Genes linked to absorption, distribution, metabolism, and excretion processes (ADME) play a critical role in the handling of pharmaceutical compounds (4). They govern metabolic pathways, substance translocation, and purification mechanisms within the body. Variations in ADME-RGs are linked to cancer development and treatment responses. Moreover, their expression patterns in tumors are believed to influence patient survival rates (5). In various cancer types, ADME-RGs have been identified as valuable prognostic markers and therapeutic targets (6–8). Although ADME-RGs are suspected to play a role in BRCA (9), their exact functions and mechanisms remain unclear, necessitating further investigation into their involvement.

This study employed bioinformatics approaches to identify ADME-related prognostic genes in BRCA and constructed a risk model based on these genes to evaluate survival differences among BRCA patients in various risk cohorts. Additionally, functional enrichment, immune infiltration, regulatory network, and drug sensitivity analyses were conducted to explore the mechanisms of action of these prognostic genes in BRCA patients.

## 2 Materials and methods

### 2.1 Data collection

BRCA-related data were obtained from the UCSC-Xena database, comprising 1,217 tissue samples, including 1,104 BRCA (tumor) and 113 normal samples, which were used as the training set. From this group, 1,082 BRCA patients with comprehensive survival and gene expression data were selected for survival analysis. The dataset labeled GSE42568, utilizing platform GPL570, contains a total of 104 BRCA specimens, which include 82 cases of invasive ductal carcinoma, 17 of invasive lobular carcinoma, and 5 samples classified under different tumor categories, alongside 17 samples from healthy breast tissues. To confirm these findings, additional datasets from the GEO repository, GSE20685 with 327 samples of breast cancer, and GSE21653 encompassing 265 BRCA specimens, 245 of which have comprehensive survival data, were acquired. Additionally, 298 ADME-RGs were identified from the literature (8).

### 2.2 Differential expression analysis

In the course of this research, version 1.38 of the DESeq2 software suite was employed to identify differentially expressed genes (DEGs) between BRCA and normal groups in the training set (10). The selection criteria for DEGs included an adjusted P-value < 0.05 and a |log2fold change (FC)| > 1. Volcano plots were created using ggplot2 (v. 3.4.4), and heatmaps were generated using Complex Heatmap (v. 2.14.0) (11, 12).

### 2.3 Identification and functional analysis of candidate genes

Candidate genes were obtained by intersecting DEGs and ADME-RGs using the Venn Diagram package (v 1.7.3) (13). Researchers investigated the functional importance and molecular routes involved in the progression of BRCA by leveraging Gene Ontology (GO) and Kyoto Encyclopedia of Genes and Genomes (KEGG) to perform enrichment studies. These studies utilized clusterProfiler software (version 4.7.1.003), setting a significance cutoff at a *P*-value of less than 0.05 (14). Furthermore, to shed light on the interactions between proteins, the STRING resource (available at https://string-db.org/, with an interaction score threshold of over 0.4) was employed. The ensuing protein-protein interaction (PPI) networks were then depicted with the aid of Cytoscape application, version 3.9.1 (15).

### 2.4 Construction and validation of a risk model

The survival package includes a function called coxph, version 3.5.3 (https://www.R-project.org/) and was employed to perform univariate Cox regression analysis on the selected candidate genes, aiming to identify those associated with prognosis (hazard ratio [HR] ≠ 1, *P* < 0.05). The methodology persisted, employing the glmnet package (version 4.1-4) to execute LASSO regression on the predictive genes. This step focused on genes that met the proportional hazards (PH) assumption test (*P* > 0.05) (16). Using these prognostic genes as a foundation, a risk model was subsequently developed. The model was constructed using the following equation: Risk score = $\sum i_{=1}^{n}\mathrm{\&x03B2;}i\times xi$, where β represents the LASSO coefficient for each gene and x denotes the expression of prognostic genes. At the same time, due to the significant difference in the number of patients between the training and validation sets, in order to avoid the impact of outliers and skewed data on the results (17), the median cutoff value of the risk score was used as the measure for high- and low-risk groups (18, 19). This provided a stable cutoff point for the classification of high- and low-risk categories for patients in both the training and validation sets. Survival differences between these groups were examined using Kaplan-Meier (K-M) survival curves generated using the Survminer package (v 0.4.9) (https://CRAN.R-project.org/package=survminer). To evaluate the risk model’s efficacy, Receiver Operating Characteristic (ROC) curves were constructed using the survivalROC package (v 1.18.0) (https://CRAN.R-project.org/package=survivalROC). The model’s performance was further verified on a separate independent dataset. To conclude, a heatmap was created to visually represent and compare the expression patterns of the prognostic genes across the identified risk groups.

### 2.5 Construction of nomogram

The training dataset underwent various statistical analyses to determine independent prognostic factors encompassing both the risk score and clinical parameters, including age and T/N/M stage (*P* < 0.05). The study included single-variable Cox regression, tests for proportional hazards (PH), and Cox regression involving multiple variables. To predict the survival chances at 3, 5, and 7 years for patients with BRCA, a prediction tool was created by applying the independent predictors discovered. The nomogram’s performance was evaluated through decision curve analysis (DCA) and receiver operating characteristic (ROC) curve analysis. The Survminer package was employed to create Kaplan-Meier survival curves, enabling the comparison of outcomes across various clinical characteristics and examination of survival disparities (*P* < 0.05).

### 2.6 Function analysis of prognostic genes

We utilized the clusterProfiler package to perform gene set enrichment analysis (GSEA) on BRCA patients across varying risk levels, aiming to uncover critical biological processes and pathways. By employing the DESeq2 package, we pinpointed genes that exhibit varying levels of expression between distinct risk groups. Following this, the log2 fold-change (log2FC) scores of these differentially expressed genes (DEGs) were calculated and ordered. The analysis employed c2.cp.kegg. v2023.1 gene set, while the Hs.symbols gene set from the Molecular Signatures Database (MSigDB, https://www.gsea-msigdb.org/gsea/msigdb) served as the background set for GSEA. The enrichment plot package (v1.18.0) was utilized to depict the five most prominent pathways (*P* < 0.05). To create a gene-gene interaction (GGI) network, GeneMANIA (https://genemania.org/) was utilized to identify genes functionally related to prognostic genes. Furthermore, the GOSemSim package (v. 2.24.0) was used to assess the functional similarity of prognostic genes using GO terms (https://guangchuangyu.github.io/software/GOSemSim). For this analysis, semantic similarity scores were calculated using the mgeneSim function.

### 2.7 Immune infiltration analysis

The CIBERSORT algorithm was employed to assess the presence of 22 common immune cell types in the training set, aiming to investigate immune cell infiltration differences between risk groups. Samples with *P* values exceeding 0.05 were omitted from the analysis. To identify statistically significant disparities in the immune cell populations between the risk cohorts for the remaining samples, we applied the Wilcoxon test. (*P* < 0.05). To evaluate the relationships between differentially abundant immune cells and prognostic genes, Spearman’s correlation analysis was conducted, with significance defined as |cor| > 0.3 and *P* < 0.05.

In addition, the Wilcoxon test was employed to calculate and contrast the immune, stromal, and ESTIMATE scores across the different risk groups (*P* < 0.05). Subsequently, the results were visually represented through graphs created with the ggplot2 package.

### 2.8 Examination of the efficacy of immune-checkpoint blockers and their responsiveness to medicinal compounds

To assess differences in the 14 ICIs previously reported in the literature between risk groups (20), the Wilcoxon test was conducted, and significant ICIs were identified (*P* < 0.05). Correlations between risk scores, prognostic genes, and differential ICIs were further explored *via* Spearman analysis using the corrplot package (v. 0.92) (https://github.com/taiyun/corrplot).

To examine variations in drug sensitivity among the risk groups, the GDSC database (https://www.cancerrxgene.org/) was used to obtain potential BRCA drugs and their corresponding half-maximal inhibitory concentration (IC50) values. The pRRophetic package (v 0.5) was employed to estimate IC50 values for each tumor sample in the training set (21). The Wilcoxon test was then employed to examine the disparities in IC50 values across the various risk categories (*P* < 0.05). A boxplot was created to display the top 10 drugs ranked according to their *p*-values.

### 2.9 Regulatory network and expression validation of prognostic genes

To investigate the regulatory mechanisms of the prognostic genes in BRCA, microRNAs (miRNAs) targeting these genes were predicted using miRDB (https://www.mirdb.org) and miRWalk (http://mirwalk.umm.uni-heidelberg.de). Crucial microRNAs were pinpointed through the process of finding the common miRNAs forecasted by the two databases. Long non-coding RNAs (lncRNAs) targeting these key miRNAs were predicted using the ENCORI database (http://starbase.sysu.edu.cn/). A regulatory network was established and depicted using the Cytoscape software, highlighting the interplay between crucial miRNAs, lncRNAs, and prognostic genes. The expression levels of prognostic genes in the BRCA and normal tissue groups within the training set (TCGA-BRCA) were compared using the Wilcoxon test, with statistical significance set at *P* < 0.05. This dataset encompassed 1217 samples, with 1104 disease cases and 113 normal controls. Boxplot illustrations were generated through the ggplot2 R package to depict the results.

### 2.10 Expression characteristics and functional enrichment analysis of prognostic genes in different subtypes of BRCA

To clarify the role of prognostic genes in different molecular subtypes of BRCA (human epidermal growth factor receptor 2-positive (HER2^+^) and triple-negative breast cancer (TNBC)), the following analysis process was adopted: BRCA clinical case information and gene expression data were extracted from the TCGA database using the R package “TCGAbiolinks” (v2.30.4) (22), and the samples were classified into HER2^+^ and TNBC subtypes according to clinical standards. A baseline analysis of the distribution characteristics of prognostic genes and clinical indicators (gender, age at diagnosis, T/N/M stage, survival time, and survival status) in the two subtypes was conducted using the R package “tableone” (v0.13.2) (https://CRAN.R-project.org/package=tableone) to test the statistical significance of the differences between the groups. The high- and low-expression groups were divided based on the median value of the prognostic gene expression within each subtype, and box plots were drawn to analyze the differences in gene expression within the subtypes. Based on the data of the high- and low-risk groups of each subtype in the TCGA–BRCA training set, gene screening differences were identified and log2FC values were calculated using the DESeq2 package (v1.40.2) (10). The log2FC values were sorted, and the clusterProfiler package (v4.8.1) (14) was used to conduct GSEA enrichment analysis with reference to the “c2. Cp. Kegg. V7.1. Symbols. GMT” gene sets in the MSigDB database. Pathways with *P* < 0.05 were screened, and the top five enrichment pathways are presented.

### 2.11 Quantitative reverse transcription polymerase chain reaction, Western blot, and immunohistochemical analysis of prognostic genes

In the research presented here, the MCF-7 and T47D cell lines served as models for an in-depth evaluation of gene prognostication across different BRCA variants. The non-tumorigenic mammary epithelial cell line MCF-10A was employed as a control for comparative analysis. Quantitative reverse transcription polymerase chain reaction (qRT-PCR) and western blot (WB) analyses were employed to evaluate the mRNA and protein expression levels of six prognostic genes in MCF-7, T47D, and MCF-10A cells. Total RNA was extracted from the three cell lines and subsequently transcribed, followed by PCR amplification using corresponding primers (**Supplementary Table S1**). The primer sequence table is shown in **Table 1**. The WB experiments were performed in triplicate, and the most representative result was selected for presentation. The main reagents used by WB are detailed in **Supplementary Table S2** of **Supplementary Materials**.

**Table 1:** Primers of the real-time reverse transcription-polymerase chain reaction.

Gene	Forward primer sequence (5’-3’)	Reverse primer sequence (5’-3’)
GSTM2	TGTGCGGGGAATCAGA AAAGG	CTGGGTCATAGCAGAG TTTGG
ADHFE1	TGGACTTTCACCTTCT GGGAA	GGAGAGGTTCTTGTCTGT CATCA
ALDH2	ATGGCAAGCCCTATGT CATCT	CCGTGGTACTTATCAGCCCA
NOS1	TTCCCTCTCGCCAAAG AGTTT	AAGTGCTAGTGGTGTCGATCT
ATP7B	GCCAGCATTGCAGAAG GAAAG	TGATAAGTGATGACGGCCTCT
ALDH3A1	TGGAACGCCTACTATG AGGAG	GGGCTTGAGGACCACTGAG

Tissue samples embedded in paraffin were collected from a randomly chosen cohort of eight individuals diagnosed with BRCA that required surgical intervention (**Supplementary Table S3**). Slides exhibiting optimal staining were chosen for presentation along with the same antibodies (**Supplementary Table S2**).

Each patient’s normal epithelial tissue adjacent to the neoplasm served as a control sample. The criteria for choosing the samples included the identification of cancerous tissue by standard immunohistochemical (IHC) staining methods, while tissue deemed normal and situated next to the cancerous area was characterized as being situated at least 1 mm from the cancerous cells and exhibiting no signs of cancer upon standard IHC staining analysis. The determination of a positive outcome necessitates a comprehensive assessment incorporating multiple variables. These crucial factors include the magnitude of staining intensity, precise localization of staining patterns, degree of non-specific background interference, spatial arrangement of cellular components, and reproducibility of findings across repeated experiments. This multifaceted analytical approach is fundamental for ascertaining whether the results can be definitively categorized as positive.

### 2.12 Statistical analysis

The R programming environment (v 4.2.2) (https://www.R-project.org/) was utilized to perform all statistical analyses. Group differences were evaluated using the Wilcoxon test, with statistical significance set at *P* < 0.05.

## 3 Results

### 3.1 Pinpointing potential genes and associated biological routes

**Figure 1:**
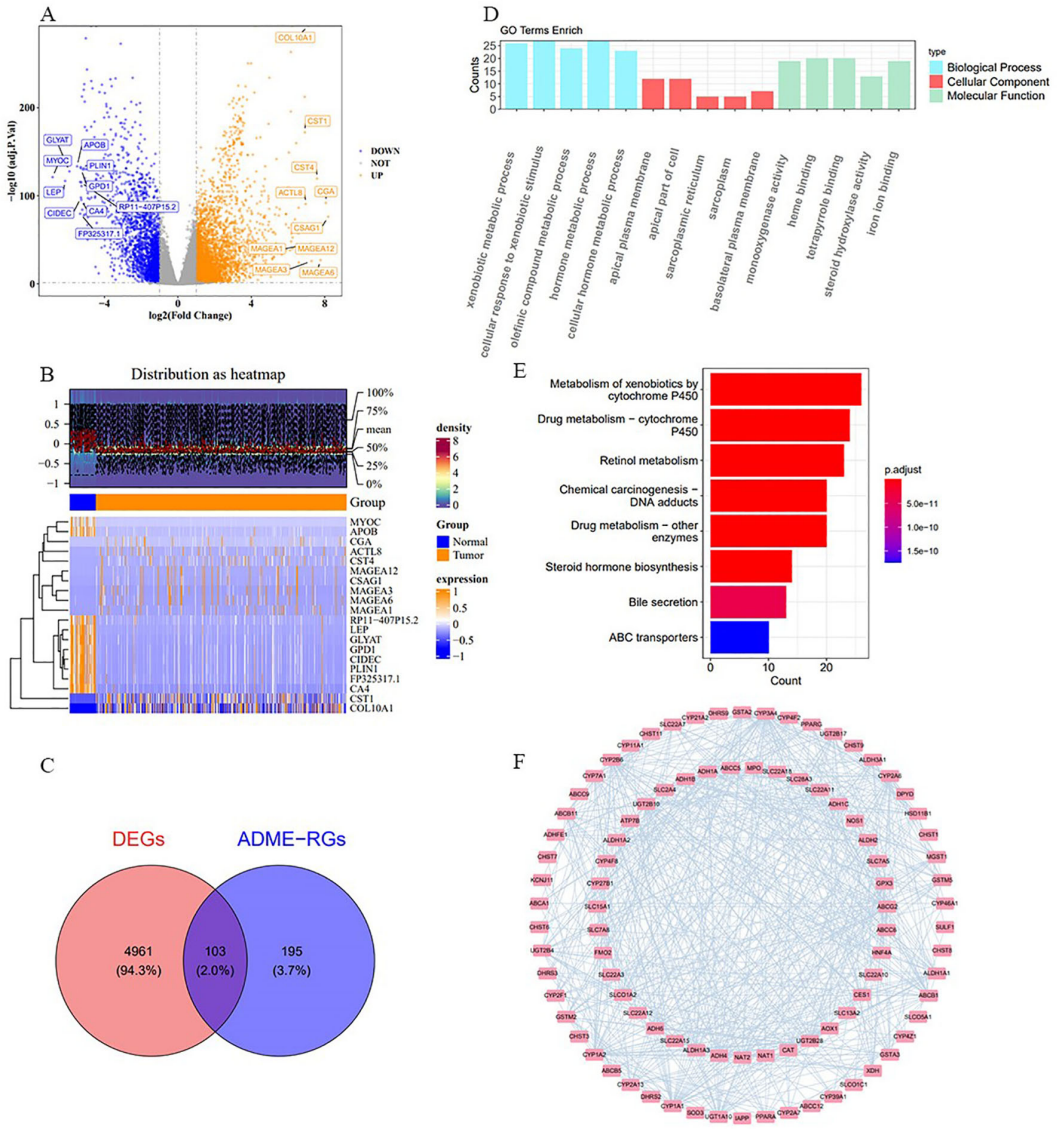
Procedure for identifying candidate genes and their associated biological functions. **(A)** The volcano plot illustrates 3,052 up-regulated (yellow) and 2,012 down-regulated (blue) genes. **(B)** Heatmap displays the distribution of DEGs, highlighting the top 10. **(C)** A total of 103 candidate genes were identified by intersecting 5,064 DEGs with 298 ADME-RGs. **(D, E)** GO and KEGG enrichment analyses of the candidate genes. **(F)** PPI network of 103 candidate genes. The nodes in the graph represent candidate genes, the edges represent interactions between genes.

For the study, we utilized the DESeq2 tool within the R framework to detect genes with significant expression differences (DEGs) when contrasting afflicted individuals with those in good health, focusing on the comparison of cancerous and non-cancerous specimens from the TCGA-BRCA data collection. The analysis revealed 5,064 DEGs, with 3,052 showing increased expression and 2,012 exhibiting decreased expression (**Figure 1A**). The top ten DEGs are shown in **Figure 1B**. By intersecting these 5,064 DEGs with 298 ADME-related genes (ADME-RGs), 103 candidate genes were identified for further investigation (**Figure 1C**). Following this, the bioinformatics tool “clusterProfiler” within the R programming environment was utilized to investigate the roles and pathways associated with these 103 potential genes in the progression of breast cancer via enrichment analysis of GO and KEGG. GO enrichment analysis identified 344 terms primarily linked to xenobiotic metabolic processes, apical plasma membrane localization, and monooxygenase activity (**Figure 1D**). Simultaneously, analysis revealed enrichment in 26 KEGG pathways, predominantly associated with the metabolism of xenobiotics through the cytochrome P450 system and the processing of drugs involving the same cytochrome P450 pathway (**Figure 1E**). These routes are linked to an elevated tumor mutation burden (TMB) and less favorable outcomes for BRCA patients. A PPI network was established, comprising 103 nodes and 625 connections (average node degree = 12.1, average local clustering coefficient = 0.482, *P* < 1 × 10^-16^). In this interconnected system, ABCA1 was connected to MPO, CYP46A1, and PPARG, while ADH1A was linked to DHRS3, CYP3A4, and ADH4 (**Figure 1F**). This network visualization allowed us to observe protein-level interactions among the differentially expressed ADMERGs.

### 3.2 GSTM2, ADHFE1, ALDH2, NOS1, ATP7B, and ALDH3A1 were selected as prognostic genes

**Figure 2:**
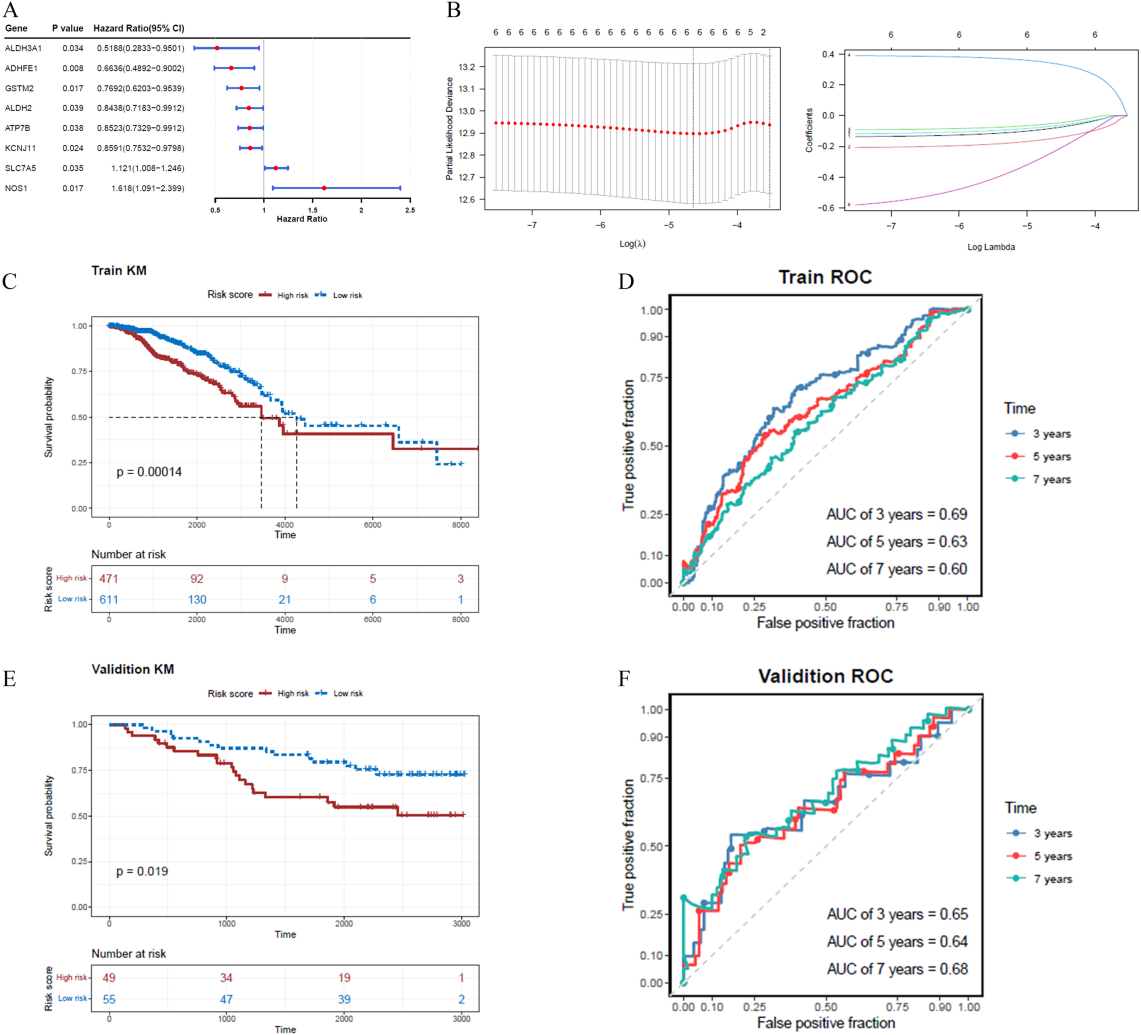
A risk model incorporating six genes was developed and validated for predicting BRCA prognosis, with the risk score calculated as follows: Risk score = $\sum \;i_{=1}^{n}\mathrm{\&x03B2;}i\times xi$. **(A)** The forest plot highlights two risk genes and six protective genes. **(B)** LASSO regression analysis identified six prognostic genes based on the optimal lambda value. **(C, D)** In the training set, the K-M survival curve demonstrated significant differences in prognosis between high- and low-risk cohorts, with corresponding ROC curves for 3, 5, and 7 years. **(E, F)** In the validation set, the K-M survival curve similarly reflected divergent prognoses between the two cohorts, accompanied by ROC curves for 3, 5, and 7 years.

Eight ADME-related prognosis genes were confirmed using univariate Cox regression analysis. The forest plot revealed that SLC7A5 and NOS1 were risk genes (HR > 1), whereas GSTM2, ADHFE1, ALDH2, ATP7B, ALDH3A1, and KCNJ11 were protective genes (HR < 1) (**Figure 2A**). Among these, six genes, GSTM2, ADHFE1, ALDH2, NOS1, ATP7B, and ALDH3A1, passed the PH assumption test (*P* > 0.05) (**Table 2**). LASSO regression analysis further narrowed the selection to six prognostic genes (GSTM2, ADHFE1, ALDH2, NOS1, ATP7B, and ALDH3A1) at lambda(min) = 0.009640588 (log(lambda) = -4.641773) (**Figure 2B**).

**Table 2:** This table presents the gene names, Chi-square test results (chisq), degrees of freedom (df), and *P*-values for the eight genes.

Genes	chisq	df	*P*-value
GSTM2	1.423047003	1	0.232902532
ADHFE1	2.987880219	1	0.083889918
KCNJ11	8.444923291	1	0.003660639
ALDH2	0.937661259	1	0.332880033
NOS1	2.194863889	1	0.13847144
ATP7B	2.428128624	1	0.119175094
SLC7A5	4.620243563	1	0.031596751
ALDH3A1	0.061019045	1	0.804892488

The findings indicate that GSTM2, ADHFE1, ALDH2, NOS1, ATP7B, and ALDH3A1 passed the PH assumption test.

A risk model was constructed using these ADME-related prognostic genes and the risk scores for patients with BRCA were calculated. The study participants were categorized into two groups, high- and low-risk, using the median value of -0.5596584 as the threshold. The K-M survival analysis demonstrated longer survival times for patients in the low-risk group, while the ROC analysis showed area under the curve (AUC) values consistently above 0.6 (**Figures 2C, D**). For the validation dataset, -0.8065118 was identified as the optimal risk score threshold, which effectively divided BRCA patients into two distinct groups. The K-M analysis results obtained from the validation set were aligned with those observed in the training set (**Figures 2E, F**; **Supplementary Figures S1A**, **S2A**). **Figure 2F** shows ROC curves for 3, 5, and 7 years, although our external validation set GSE20685 was for 7, 9, and 11 years (**Supplementary Figure S1B**), and GSE21653 was for 1, 3, and 5 years (**Supplementary Figure S2B**), their AUC value was greater than 0.7, which proved that our model was valid. We lengthened the prediction timeframe after reviewing the validation outcomes.

Moreover, the mortality risk curve and survival status charts indicated that higher risk scores were correlated with increased death rates (**Supplementary Figures S1C, D**, **S2C, D**). Analysis of prognostic gene expression between risk groups showed that GSTM2 and ADHFE1 were more highly expressed in the low-risk cohort (**Supplementary Figures S1E**, **S2E**, **S3**, **S4**).

### 3.3 Nomogram had excellent predictive efficacy

**Figure 3:**
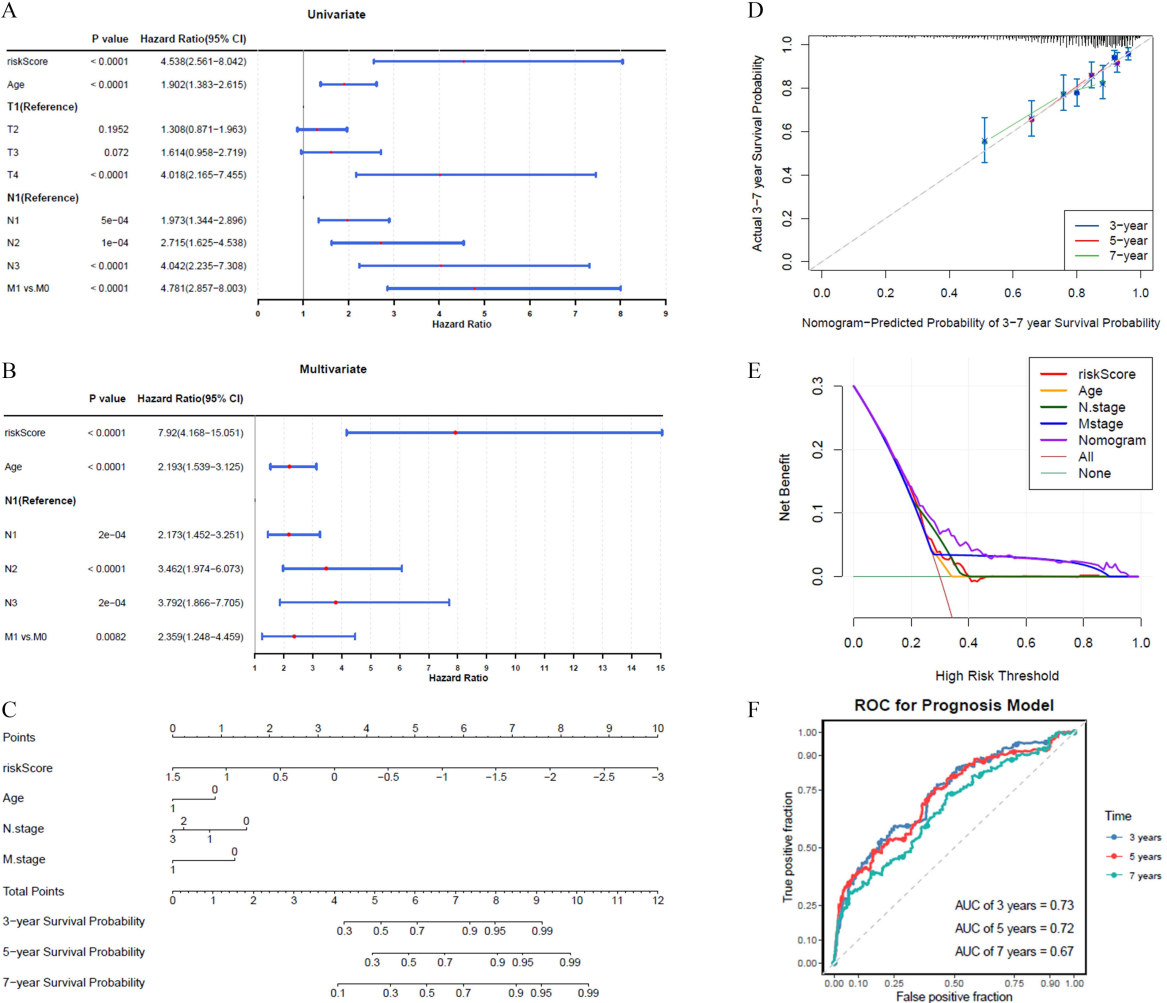
The abilities of nomograms to predict prognosis of BRCA. **(A)** Univariate Cox analysis showed risk score, age, and T/N/M stage as prognosis-related factors. **(B)** Multivariate Cox analysis showed risk score, age, and N/M stage as independent prognostic factors. **(C, D)** The nomogram showed good predictive performance for survival probability, calibration curve had higher coincidence with ideal curve. **(E)** DCA curve indicated that nomogram has higher overall prediction effect. **(F)** AUC value demonstrated that nomogram has an effective clinical predictive capability in 3, 5, and 7 years.

The results of the univariate Cox regression analysis indicated that the prognosis of BRCA could be significantly predicted by the risk score, patient age, and T/N/M stage, as evidenced by *p*-values less than 0.05 (**Figure 3A**). Among these, all factors except T stage passed the PH assumption test and were included in further analysis (*P* > 0.05). Further analysis employing the multivariate Cox model indicated that the prognostic risk score, patient age, and N/M stage were shown to be independent prognostic determinants (*P* < 0.05) (**Figure 3B**).

A predictive model was developed to calculate the survival likelihood for individuals diagnosed with BRCA. (**Figure 3C**). The calibration curve demonstrated strong alignment with the ideal curve (**Figure 3D**), while the DCA curve indicated that the overall predictive performance of the nomogram surpassed that of the individual factors (**Figure 3E**). The nomogram demonstrated strong clinical predictive accuracy, as evidenced by AUC scores of 0.73, 0.72, and 0.67 at 3, 5, and 7 years, respectively (**Figure 3F**).

Additionally, K-M survival analysis showed that when high- and low-risk categories were compared across various clinical indicators, individuals within the low-risk classification demonstrated markedly improved longevity in both the above 60 and at or below 60 years age cohorts, as depicted in **Supplementary Figure S5**.

### 3.4 Enriched pathways and function-related genes of prognostic genes

**Figure 4:**
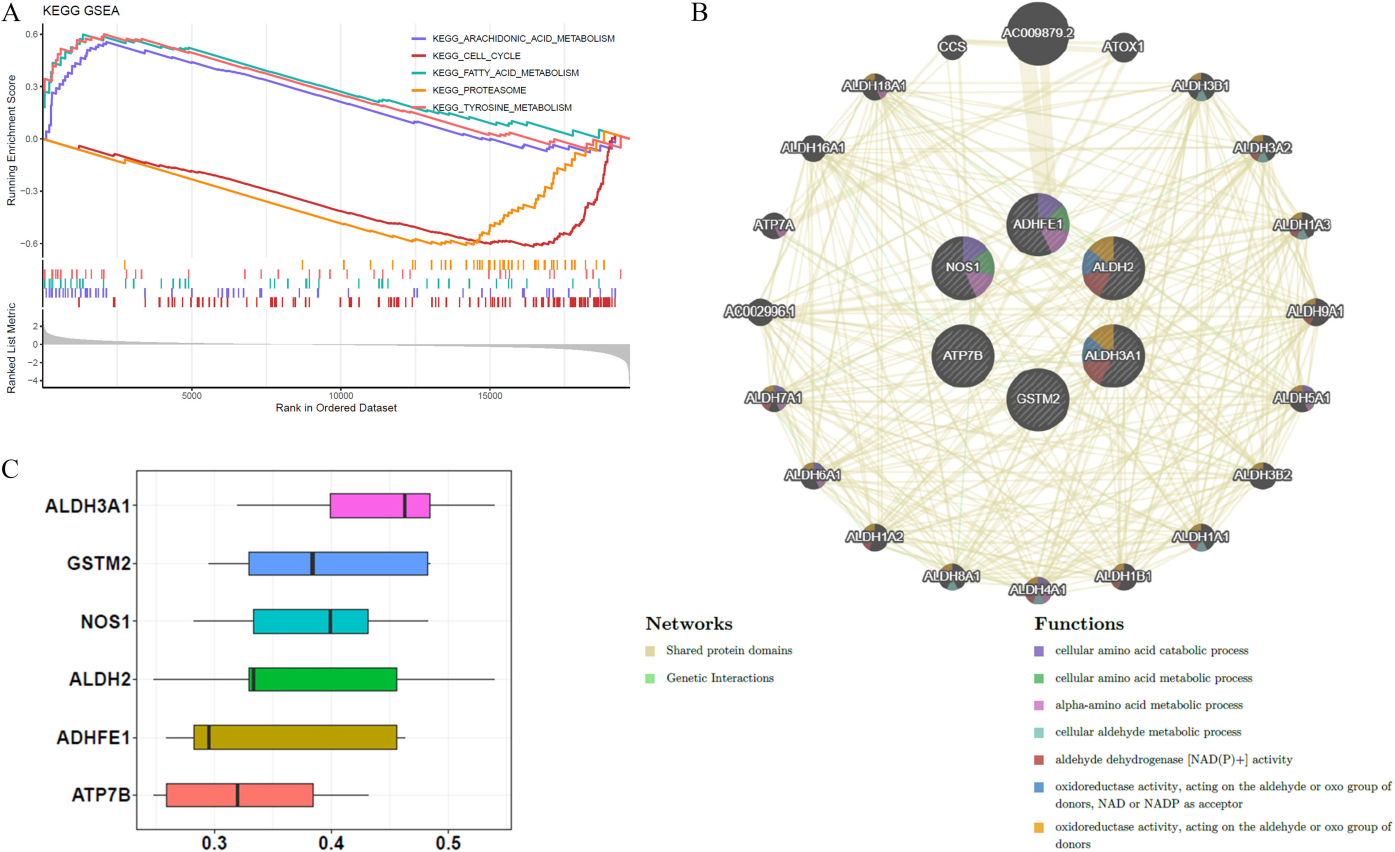
Biological functions and pathways associated with the six prognostic genes. **(A)** KEGG GSEA enrichment analysis identified the top 5 pathways related to prognostic genes. **(B)** GGI networks demonstrated interactions among these prognostic genes, involving multiple essential biological processes. **(C)** Friends analysis confirmed that ALDH3A1 exhibited higher functional similarity compared to the other five prognostic genes.

GSEA was conducted to investigate biological functions and pathways associated with BRCA. *P*-values indicate that the most statistically significant pathways were arachidonic acid metabolism, cell cycle regulation, fatty acid metabolism, proteasome function, and tyrosine metabolism, ranking as the top five most important (**Figure 4A**). GeneMANIA analysis identified 20 genes functionally linked to ADME-related prognostic genes, and a GGI network was constructed, highlighting interactions such as AC009879.2-ADHFE1, ALDH2-ALDH3B1, and NOS1-CCS. The results of this examination suggest that ADHFE1 plays a role in various essential biological functions, including the breakdown and metabolism of cellular amino acids, as well as the metabolism of alpha-amino acids (**Figure 4B**). Moreover, ALDH3A1 demonstrated the highest functional similarity among the prognostic genes, as shown by the Friends analysis (**Figure 4C**).

### 3.5 Diverse immune microenvironment between high and low risk cohorts

**Figure 5:**
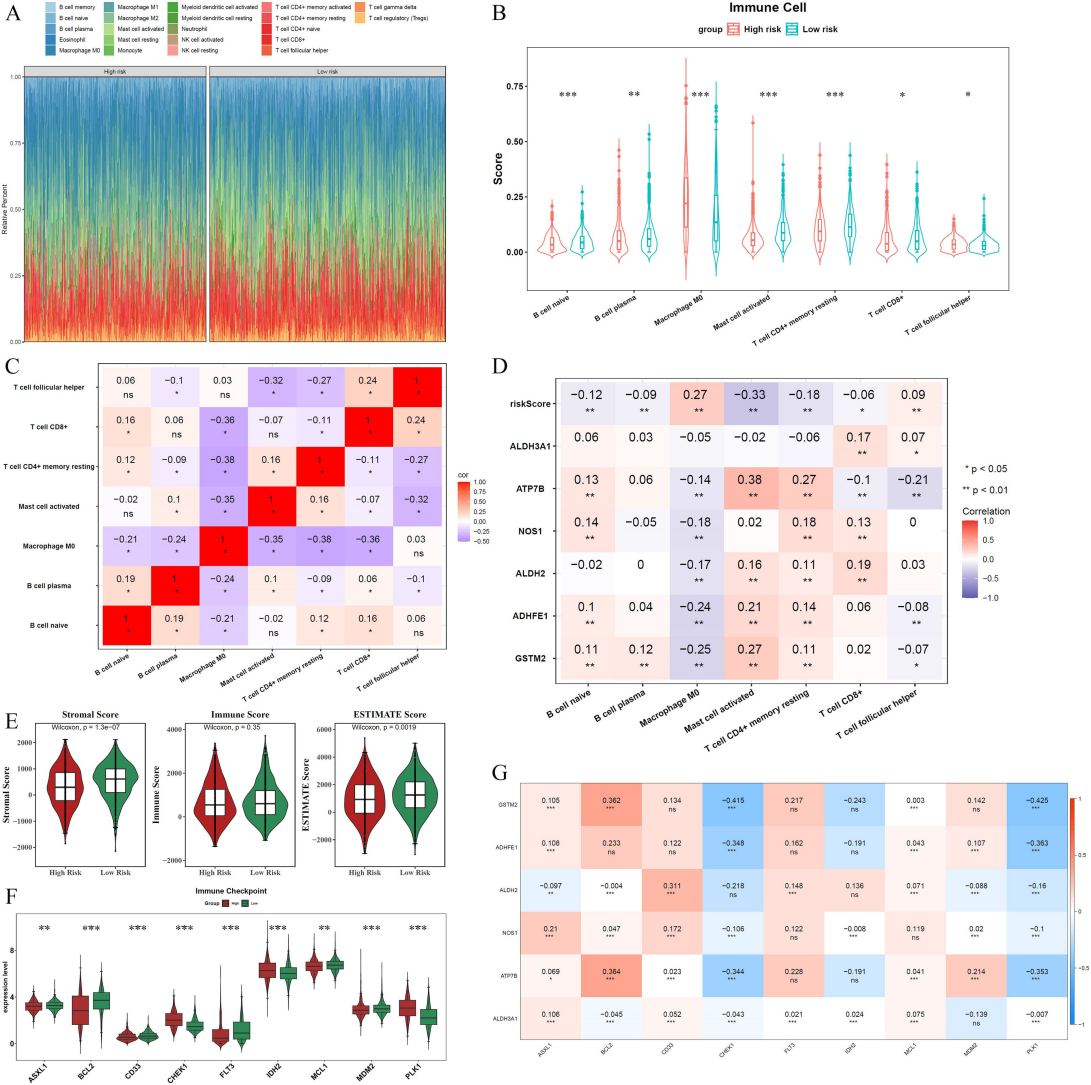
Immune microenvironment landscape differences between high- and low-risk groups. **(A)** Infiltration abundance of 22 immune cell types across the two risk cohorts. **(B)** Seven immune cell types showing significant differences between the two groups. **(C)** Spearman correlation analysis illustrates the relationships among the seven immune cell types. **(D)** Correlation analysis between differentially expressed immune cells, prognostic genes, and the risk score. **(E)** Stromal and ESTIMATE scores were significantly higher in the low-risk group. **(F)** Nine types of ICIs exhibited significant differences between the risk groups. **(G)** Correlations between the six prognostic genes and ICI efficacy. Asterisks represent statistical significance (*P < 0.05; **P < 0.01; ***P < 0.001; ns: no significance).

The infiltration abundance of the 22 immune cell types in the different risk cohorts is illustrated in **Figure 5A**. Following the elimination of samples with *P* > 0.05, a Wilcoxon test was conducted to evaluate immune cell disparities between the two groups. Considerable divergence was noted among the seven types of immune cells when comparing groups at various levels of risk, including naive B cells, plasma B cells, and M0 macrophages. Except for M0 macrophages, all immune cells exhibited elevated expression levels in the low-risk group (**Figure 5B**). A significant inverse relationship between resting memory CD4+ T cells and M0 macrophages was identified through Spearman’s correlation analysis (cor = -0.38, *P* < 0.05) (**Figure 5C**). Furthermore, a correlation study examining the relationship between different immune cell types and ADME-related prognostic genes revealed that ATP7B exhibited a positive correlation with activated mast cells (cor = 0.38, *P* < 0.05). In contrast, activated mast cells demonstrated a negative correlation with the risk score (cor = -0.33, *P* < 0.05) (**Figure 5D**).

Additional examination revealed that the low-risk group exhibited significantly elevated stromal and ESTIMATE scores compared to other groups (**Figure 5E**). Moreover, nine types of ICIs, including ASXL1, BCL2, CD33, CHEK1, FLT3, IDH2, MCL1, MDM2, and PLK1, showed significant differences between the risk groups. Among these, only CHEK1, IDH2, and PLK1 were elevated in the high-risk group, whereas the remaining ICIs were higher in the low-risk group (**Figure 5F**). Additionally, most ADME-related prognostic genes were positively correlated with ICIs (cor > 0, *P* < 0.05), except for CHEK1 and PLK1, which showed negative correlations (cor < 0, *P* < 0.05). The risk score also exhibited strong associations with ICIs (**Figures 5G**, **S6**).

### 3.6 Prospective medications and regulatory network of prognostic genes

**Figure 6:**
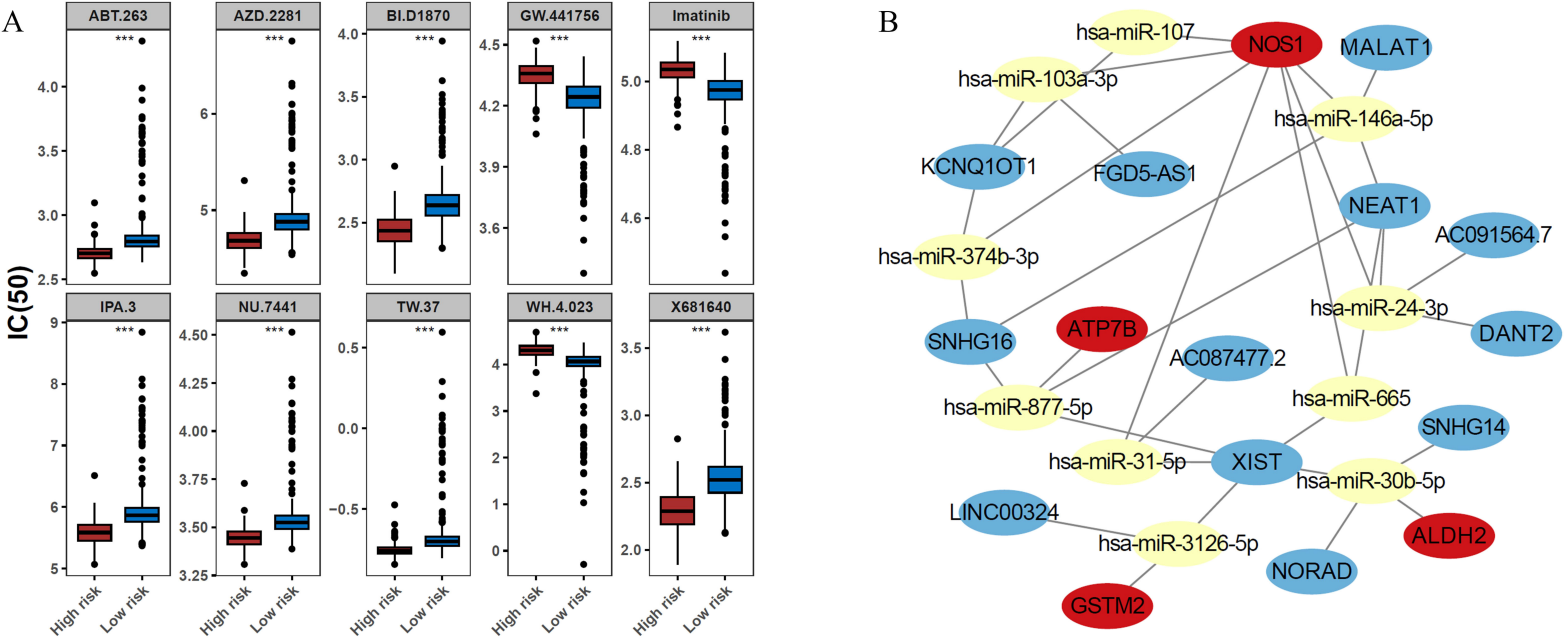
Prospective therapeutic agents and a competing endogenous RNA (ceRNA) regulatory network of the ADME-related prognostic genes. **(A)** Comparison of the half-maximal inhibitory concentration (IC50) values for the top 10 candidate drugs between the high- and low-risk groups. Drugs such as GW.441756, imatinib, and WH.4.023 exhibited lower IC50 values (higher efficacy) in the low-risk group. In contrast, the high-risk group showed increased sensitivity to the remaining seven drugs (ABT.263, AZD.2281, BI.D1870, IPA.3, NU.7441, TW.37, and X681640). **(B)** ceRNA network illustrating the potential regulatory mechanisms of the prognostic genes. The network comprises 4 prognostic genes, 10 miRNAs and 12 lncRNAs. Key regulatory axes include the regulation of NOS1 by MALAT1/SNHG16/NEAT1 via hsa-miR-146a-5p, and the regulation of ALDH2 by NORAD/SNHG14/XIST via hsa-miR-30b-5p.

To identify potential therapeutic drugs for patients with BRCA, the IC50 values of various drugs were calculated and compared between risk groups. Among the top 10 drugs with significant differences, GW.441756, imatinib, and WH.4.023 were more effective in the low-risk group, whereas the remaining seven drugs (ABT.263, AZD.2281, BI. D1870, IPA.3, NU.7441, TW.37, and X681640) showed higher sensitivity in the high-risk group (**Figure 6A**). In terms of regulatory mechanisms, a lncRNA-miRNA-mRNA regulatory network was constructed, incorporating four ADME-related prognostic genes, 10 miRNAs, and 12 lncRNAs. Within this network, MALAT1, SNHG16, and NEAT1 were found to regulate NOS1 *via* hsa-miR-146a-5p, whereas NORAD, SNHG14, and XIST regulated ALDH2 via hsa-miR-30b-5p (**Figure 6B**).

### 3.7 Analysis results of prognostic genes and related characteristics in HER2^+^ and TNBC subtypes

A total of 178 HER2+ and 122 TNBC samples were included in this study for analysis. The results of the baseline characteristic analysis (**Table 3**) showed that among the prognostic genes, there were significant differences in the expression levels of ALDH2, NOS1, ATP7B, and ALDH3A1 between the two subtypes (*P* < 0.05). Among the clinical indicators, there was a significant difference in age at diagnosis between the HER2+ and TNBC subtypes (*p* = 0.002).

**Table 3:** Baseline table of prognostic genes and clinical information characteristics among molecular subtypes.

n	level	Overall	HER2+	TNBC	*p*
		300	178	122	
GSTM2 (mean (SD))		1.12(0.81)	1.17(0.84)	1.04(0.75)	0.187
ADHFE1 (mean (SD))		1.02(0.73)	1.02(0.74)	1.02(0.73)	0.976
ALDH2 (mean (SD))		3.28(1.09)	3.46(1.03)	3.03(1.13)	0.001
NOS1 (mean (SD))		0.05(0.26)	0.03(0.06)	0.09(0.40)	0.028
ATP7B (mean (SD))		1.65(0.92)	2.06(0.89)	1.05(0.58)	<0.001
ALDH3A1(mean (SD))		0.28(0.66)	0.20(0.45)	0.39(0.87)	0.015
gender (%)	Male	4(1.3)	4(2.2)	0(0.0)	0.248
	Female	296(98.7)	174(97.8)	122(100.0)	
age (mean (SD))		56.87(12.91)	58.76(13.43)	54.11(11.63)	0.002
TNM.stage (%)	Stage I	41(13.7)	20(11.2)	21(17.2)	0.168
	Stage II	185(61.7)	107(60.1)	78(63.9)	
	Stage III	69(23.0)	48(27.0)	21(17.2)	
	Stage IV	5(1.7)	3(1.7)	2(1.6)	
OS.time (mean (SD))		682.48(781.97)	627.03(727.15)	763.39(852.29)	0.138
OS (%)	0	259(86.3)	153(86.0)	106(86.9)	0.953
	1	41(13.7)	25(14.0)	16(13.1)	

**Figure 7:**
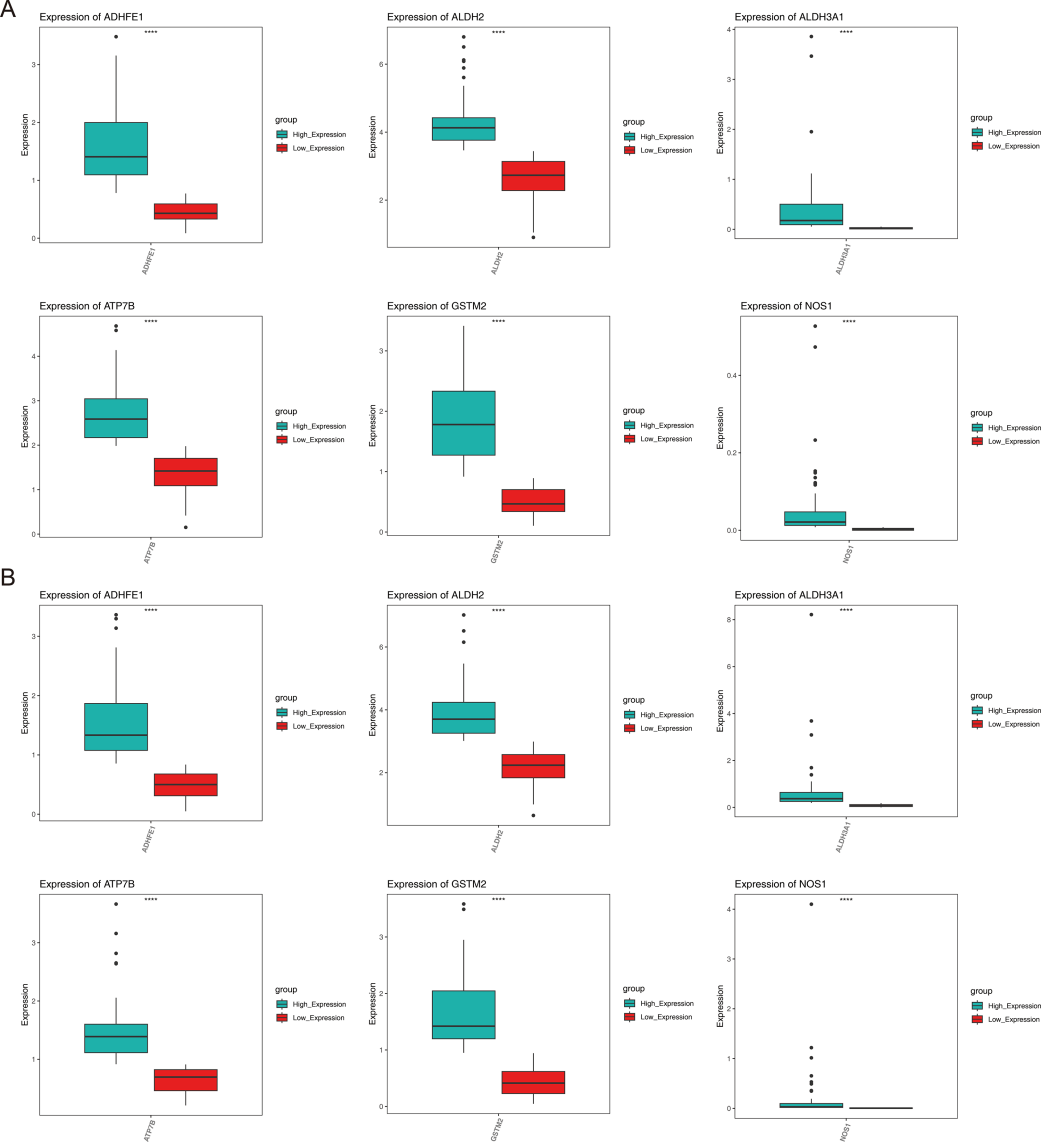
Expression of prognostic genes in different subtypes of breast cancer. **(A)** HER2+, **(B)** TNBC.

Analysis of gene expression differences showed that for all prognostic genes, there were significant differences between the high- and low-expression groups within the HER2+ and TNBC subtypes (*P* < 0.05) (**Figure 7**).

**Figure 8:**
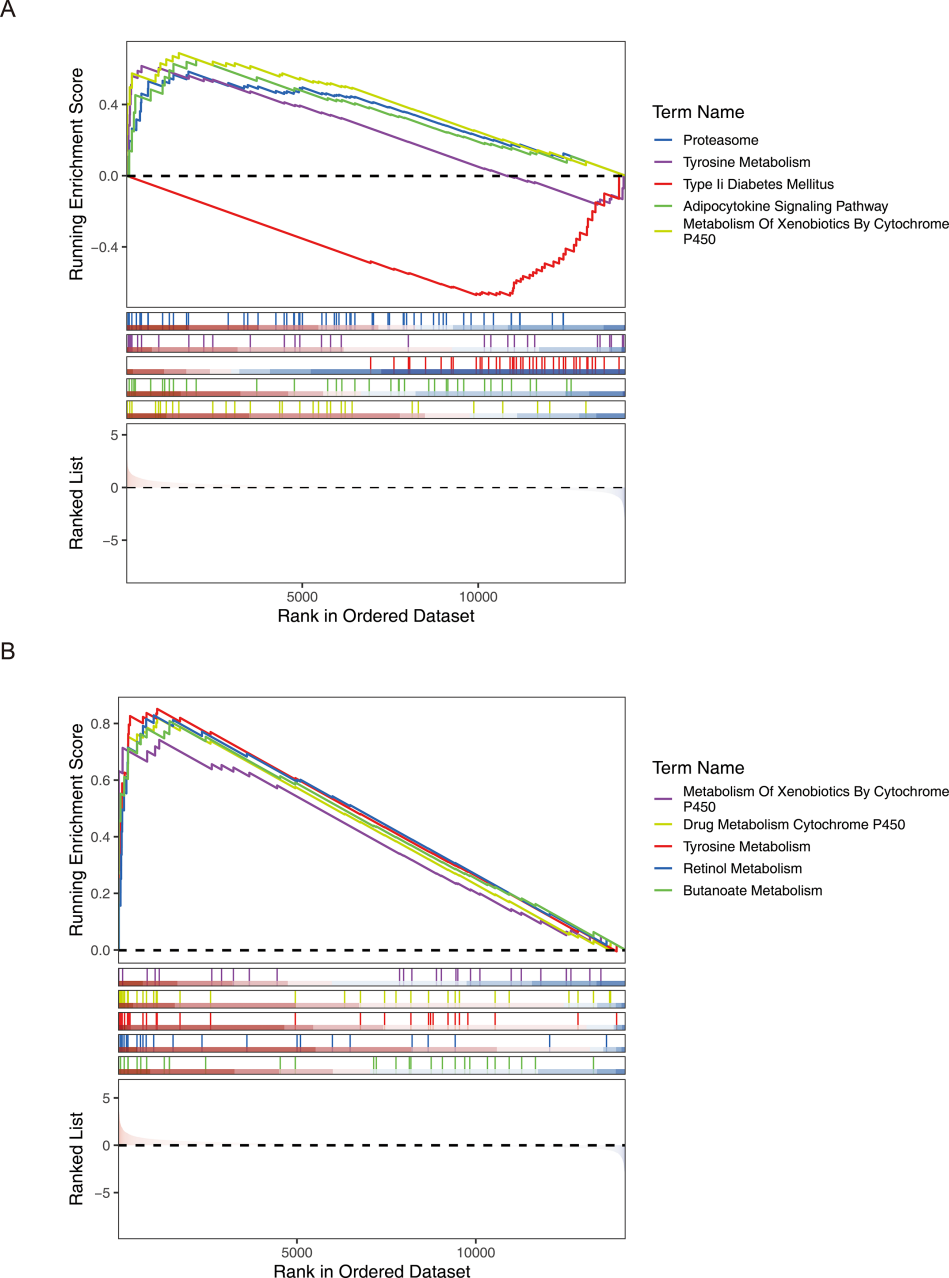
GSEA analysis revealed multiple significantly enriched pathways in the high- and low-risk groups of both HER2+ and TNBC subtypes, showing similar functional association patterns. **(A)** HER2+ subtype **(B)** TNBC subtype.

The GSEA enrichment analysis results showed that a total of 21 significant pathways were enriched in the high - risk and low - risk groups of the HER2^+^ subtype. The TOP5 pathways were Proteasome, Tyrosine Metabolism, Adipocytokine Signaling Pathway, Type II Diabetes Mellitus, and Metabolism of Xenobiotics by Cytochrome P450. A total of 30 significant pathways were enriched in the high - risk and low - risk groups of the TNBC subtype. The TOP5 pathways included heterobiomass Metabolism mediated by Cytochrome P450, Drug Metabolism of cytochrome P450, tyrosine metabolism, Retinol Metabolism, and Butanoate Metabolism (**Figure 8**). The above results indicated that prognostic genes showed consistent expression difference patterns and similar functional pathway associations in the HER2+ and TNBC subtypes, once again demonstrating the prognostic value of these genes.

### 3.8 Expression analysis of prognostic genes at different levels

The expression levels of ADME-related prognostic genes were analyzed. The boxplot results indicated that ATP7B exhibited significantly higher expression in the BRCA group, while the remaining ADME-related prognostic genes were expressed at lower levels in the BRCA group (*P* < 0.001) (**Supplementary Figure S7**).

**Figure 9:**
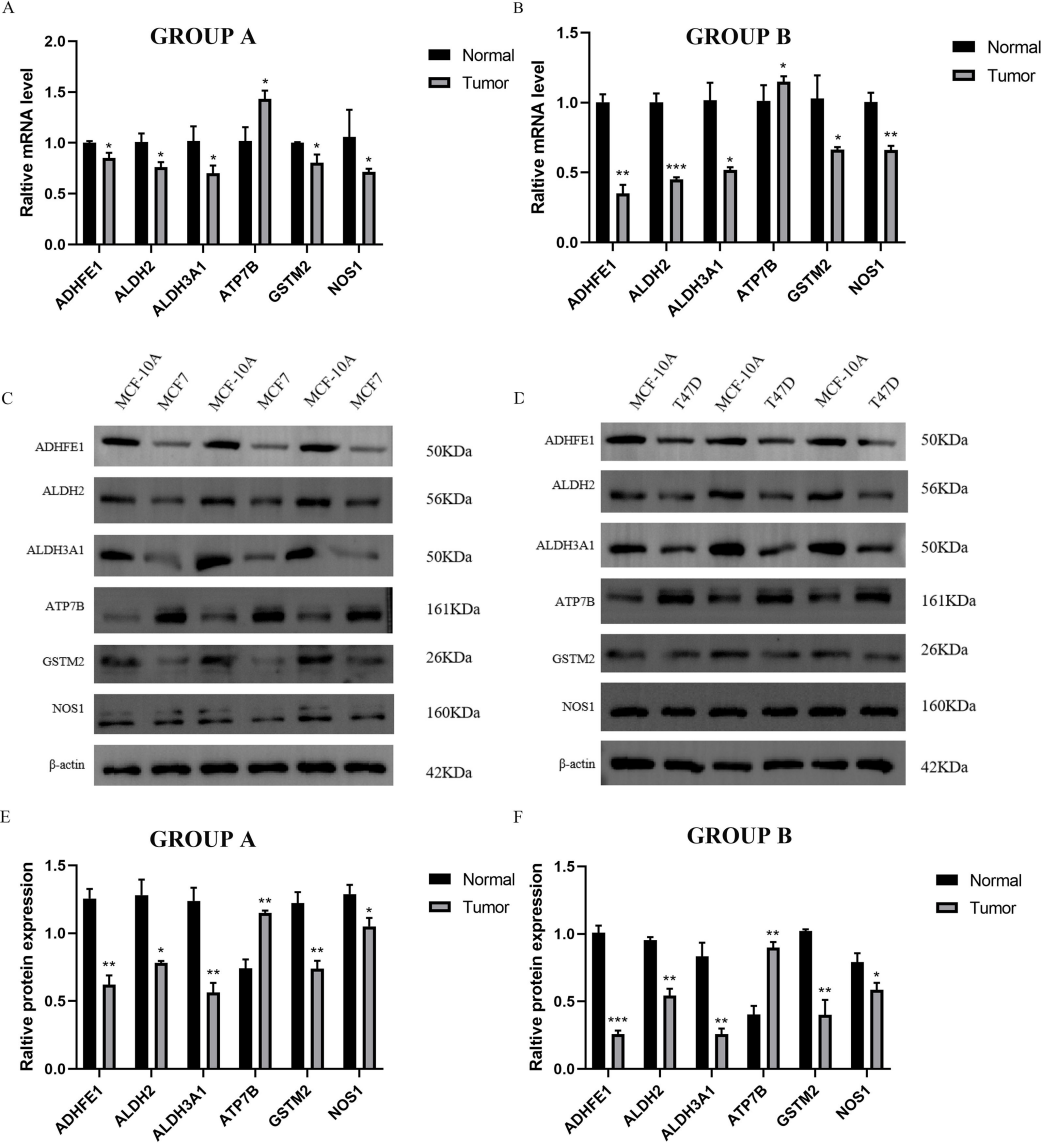
Independent verification by three cell lines. GROUP A represented MCF-7 (Tumor) and MCF-10A (Normal); GROUP B represented T47D (Tumor) and MCF-10A (Normal). **(A, B)** The qRT-PCR results of MCF-7, T47D and MCF-10A. **(C, D)** Electrophoretic maps and molecular weights of six proteins in three cell lines, MCF-7, T47D and MCF-10A. **(E, F)** The WB results of MCF-7, T47D and MCF-10A. (*P < 0.05; **P < 0.01; ***P < 0.001).

We identified six ADME-related mRNA expression levels in three cell lines by qRT-PCR, the results of **Figures 9A, B** showed that compared to MCF-10A cells, high expression of ATP7B in MCF-7 and T47D respectively (*P* < 0.05), while low expression of other five genes separately (*P* < 0.05), which high similarity to TCGA-BRCA. In addition, the same trends of six proteins expression levels in MCF-7, T47D and MCF-10A cells were observed by electrophoretic results (**Figures 9C, D**). The boxplot results also showed that ATP7B in MCF-7 and T47D cells were higher than MCF-10A (*P* < 0.05), at the meanwhile, the other five proteins (GSTM2, ADHFE1, ALDH2, NOS1 and ALDH3A1) were significantly lower in BRCA cells than normal cells (**Figures 9E, F**) (*P* < 0.05).

In summary, we further verified the gene and protein expression levels of GSTM2, ADHFE1, ALDH2, NOS1, ATP7B, and ALDH3A1 through independent external experiments using qRT-PCR and WB blot.

**Figure 10:**
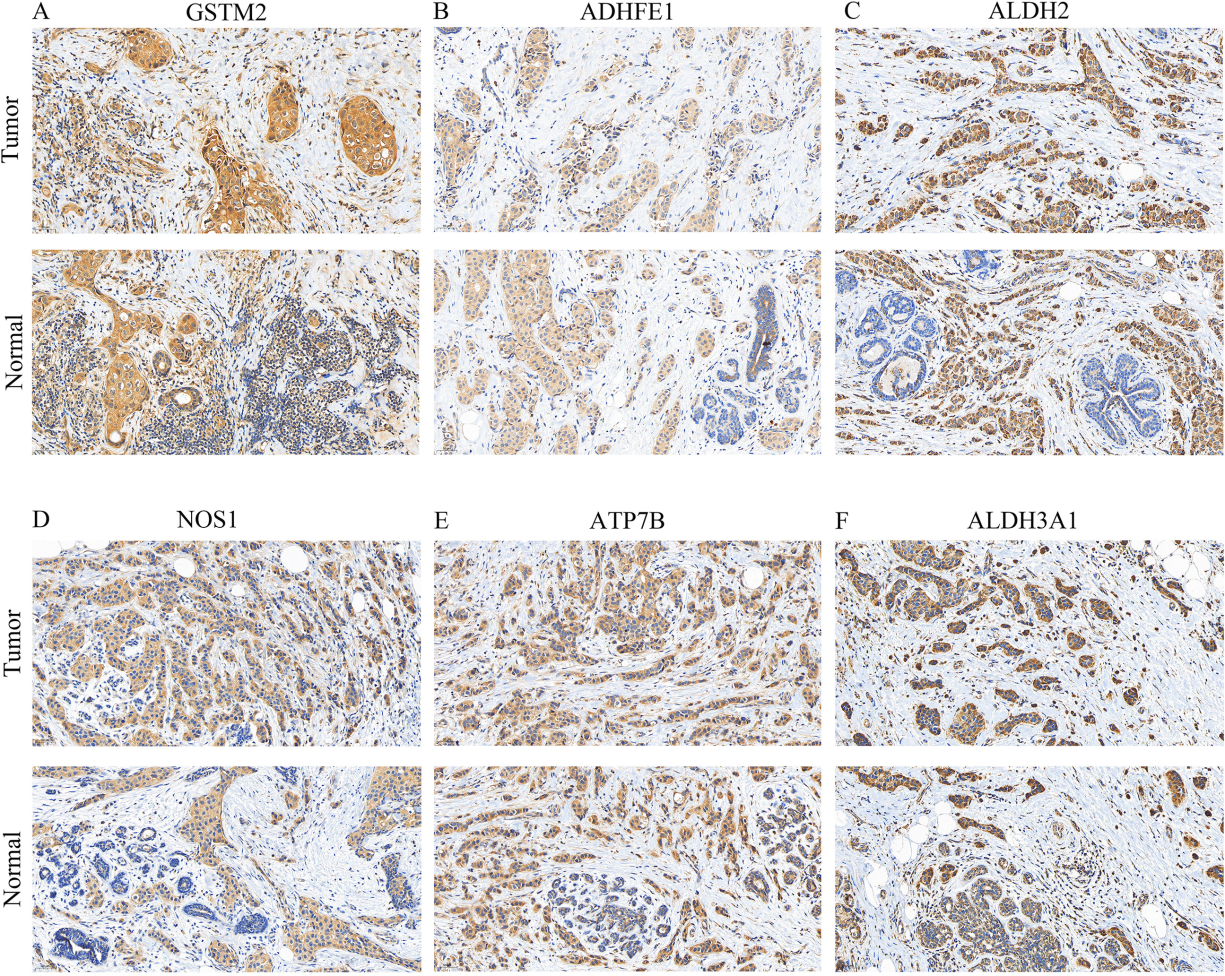
IHC staining (× 200) of prognostic gene expression. **(A–F)** Protein expression levels of GSTM2, ADHFE1, ALDH2, NOS1, ATP7B, and ALDH3A1 in BRCA and normal tissues.

Finally, the expressions of GSTM2, ADHFE1, ALDH2, NOS1, ATP7B, and ALDH3A1 were visualized by IHC. Positive results are indicated by a blue coloration in the nucleus and a brownish-yellow or brown hue for the target proteins. Additionally, the results revealed that GSTM2, ALDH2, ADHFE1, and ATP7B were especially elevated in the BRCA group (**Figures 10A-F**). This suggested that these genes might have been associated with the occurrence or progression of breast cancer.

## 4 Discussion

Research has demonstrated that variations in ADME-related genes are strongly linked to the onset, progression, and treatment of BRCA. These genes encode enzymes and transporters that play a role in metabolism and movement of foreign substances, including medications and cancer-causing agents. Additionally, variations in ADME genes contribute to differences in ADME function among individuals, which in turn affect BRCA susceptibility and the body’s response to drugs (23–25). Polymorphisms in the CYP2D6 gene impact the metabolism and activation of tamoxifen, consequently influencing its therapeutic efficacy and side effect profile during the treatment of BRCA-related cancers (26). Moreover, abnormal expression of ADME genes can promote BRCA advancement by modifying the processing and elimination of medications and cancer-causing substances (27–29). Therefore, a comprehensive investigation of the relationship between ADME-RGs and BRCA is critical. Such research will not only illuminate the pathogenic mechanisms of BRCA but will also assist in optimizing current treatment strategies and potentially uncovering novel therapeutic approaches.

After identifying genes associated with both ADME-RGs and BRCA, subsequent enrichment analysis demonstrated their participation in pathways related to xenobiotic and drug metabolism involving cytochrome P450 (**Figure 1**). This case-control investigation sheds light on the intricate dynamics between genetic susceptibility and environmental influences, providing a considerable understanding of the alterable elements of risk (30). Studies have shown that ATP-binding cassette subfamily G member 2 (Abcg2) facilitates tolfenamic acid transport, affecting its plasma concentration and tissue distribution, which may alter its pharmacological effects and toxicity (31). Moreover, a mutation in Mitochondrial Dysfunctional 1 (MDN1) was enriched in drug metabolism cytochrome P450 pathways and associated with a high tumor mutational burden (TMB) and poorer prognosis in patients with BRCA. This suggests that MDN1 mutation could serve as a prognostic biomarker and inform immunotherapy decisions for patients with BRCA (32). Given the findings of previous studies on ADME-RGs mutations in BRCA, it is hypothesized that variations in ADME genes, particularly those involved in these critical biological processes and pathways, may increase BRCA susceptibility. However, further research is required to validate these results and elucidate the underlying mechanisms.

The prognostic significance of six genes (GSTM2, ADHFE1, ALDH2, NOS1, ATP7B, and ALDH3A1) was confirmed (**Table 2**; **Figure 2**). Based on these findings, a risk model was developed that demonstrated that high-risk patients experienced significantly reduced survival durations. (*P* < 0.05). The AUC values further demonstrated strong predictive performance (**Figures 2C, D–F**). It is worth noting that six prognostic genes showed highly consistent results with transcriptome analysis in qRT-PCR and WB analysis, indicating that the expression trend of these genes was consistent at the mRNA level and protein level, further verifying the accuracy and reliability of transcriptome analysis. These genes exhibited a strong correlation with prognosis, indicating their potential as BRCA biomarkers, which is consistent with the results of previous studies. Hypermethylation of the glutathione S-transferase mu 2 (GSTM2) promoter has been identified as a potential biomarker for aggressive tumor behavior and may contribute to the progression of estrogen receptor (ER)-and progesterone receptor (PR)-negative BRCA (9). Alcohol dehydrogenase iron-containing 1 (ADHFE1), an oncogene, induces metabolic reprogramming, promoting both tumor growth and metastasis in BRCA (33). Aldehyde dehydrogenase 2 family member (ALDH2) and nitric oxide synthase 1 (NOS1) have been linked to lymph node metastasis and bone metastasis in BRCA, respectively (20, 21). A pan-cancer analysis revealed that ATPase copper transporting beta (ATP7B) negatively correlates with macrophage infiltration in BRCA and is strongly associated with prognosis, immunotherapy response, and disease progression (34). Aldehyde dehydrogenase 3 family member A1 (ALDH3A1), an enzyme involved in drug metabolism, is negatively correlated with peroxisome proliferator-activated receptor γ (PPARγ) and is implicated in cancer cell resistance to anticancer drugs (35). However, it is noteworthy that the expression of ADME-related genes may vary among different BRCA subtypes. Research findings indicated that basal-like subtype patients exhibited reduced ATP7B expression, implying that copper concentrations in the tumor tissue of individuals with basal-like breast cancer may vary from those observed in other breast cancer subtypes (36). However, this should be further researched. A nomogram was developed to assess the survival likelihood of BRCA patients, aiming to further test the risk model’s predictive capabilities. The calibration curve, DCA curve, and AUC values confirmed the high predictive accuracy of the nomogram (**Figure 3**). These findings suggest that the constructed nomogram may serve as a potential reference tool for risk assessment in patients with BRCA and could provide supportive value for predicting treatment outcomes. The robustness of the model was further confirmed using an independent external dataset. Previous studies have indicated that internal validation approaches alone cannot guarantee the quality of machine learning models, as the training data may be biased, and the validation process is inherently complex. Therefore, external validation is essential for evaluating the generalizability of predictive models (37), which highlights the rationality of the validation strategy employed in this study. To further investigate the role of prognostic genes in the different BRCA subtypes (**Table 3**), we performed expression validation and enrichment analyses. The results showed that the six ADME-related prognostic genes exhibited stable expression difference patterns in both the HER2+ and TNBC subtypes. In addition, the core-enriched pathways in the high- and low-risk groups of both subtypes were highly associated with the metabolic regulation and drug-processing functions of ADME genes. This not only confirms the universal prognostic value of these genes across subtypes but also addresses the limitations of previous prognostic models that lacked subtype-specific analyses. Given that HER2+ and TNBC subtypes differ markedly in clinical treatment strategies, our results imply that an ADME gene-based prognostic model could be applicable for risk stratification in patients with different subtypes of breast cancer. Furthermore, the subtle differences in core pathways between subtypes suggest that ADME genes may influence BRCA progression via subtype-specific molecular mechanisms, providing direction for future investigations into subtype-exclusive ADME-related therapeutic targets.

Further investigations showed that genes with prognostic value were predominantly associated with pathways involving tetraenoic acid metabolism, regulation of the cell cycle, fatty acid metabolism, degradation via the proteasome, and metabolism of tyrosine (**Figure 4A**). Li et al. suggested that heightened arachidonic acid metabolism could serve as a favorable prognostic marker in BRCA, potentially explaining the limited efficacy of cyclooxygenase inhibitors in cancer therapy. This insight offers a novel perspective on management (38). In cell cycle regulation, research demonstrated that Keratin 19 (K19) deficiency disrupts normal cell cycle progression, highlighting K19’s critical role in cell cycle control and its potential as a predictive marker for cyclin-dependent kinase (CDK) inhibitor efficacy in BRCA treatment (39). The production of fatty acids, primarily orchestrated by fatty acid synthase (FASN), is frequently upregulated and excessively active in malignant tumors, contributing to their growth and spread (40). Recent studies on fatty acid metabolism have linked its dysregulation to cancer cell invasion and diminished immune cell infiltration in male breast cancer (MBC), suggesting a poor prognosis for affected patients (41). Increasing evidence points to proteasomes as potential therapeutic targets for BRCA. For example, one study emphasized the protective role of Nuclear Respiratory Factor 1 (NRF1), which enhances proteasome gene expression in response to proteasome inhibition, indicating a possible treatment avenue for BRCA (42). A recent study found that the tyrosine-phosphorylation-facilitated interaction between Yes-associated protein 1 (YAP1) and Transcription Factor AP-2 Alpha (TFAP2A) is essential for regulating gene expression and contributes to trastuzumab resistance in HER2+ BRCA. Combining HER2 inhibition with targeting YAP1 transcriptional activity could effectively counteract trastuzumab resistance caused by non-receptor tyrosine kinase (SRC) activation (43). Few studies have examined how prognostic genes regulating these signaling pathways affect BRCA outcomes. Further research is required to understand the physiological roles and interactions of these genes in these pathways.

The observed associations among immune cell infiltration, ADME-related prognostic genes, and immune checkpoint inhibitors (ICIs) in this study provide valuable insights into the potential immune–molecular mechanisms underlying prognostic differences in BRCA. Seven immune cell types, including naïve B cells and plasma cells, were significantly enriched in the low-risk group, whereas M0 macrophages were upregulated in the high-risk group. This pattern suggests that the low-risk group may exhibit a more active adaptive immune response and a lower pro-inflammatory state, whereas the high-risk group may be characterized by aberrant macrophage polarization or the establishment of an immunosuppressive microenvironment, phenomena that are highly consistent with established antitumor immune mechanisms. Naïve B cells, for example, can differentiate into plasma cells to secrete antigen-specific antibodies and participate in humoral immunity (44), and high plasma cell infiltration is frequently associated with favorable clinical outcomes in patients with cancer (45). In contrast, M0 macrophages, as unpolarized precursors, are prone to shift toward a tumor-promoting M2 phenotype in the tumor microenvironment (46), providing a plausible explanation for the more favorable baseline prognosis observed in the low-risk group.

Furthermore, our results revealed a significant negative correlation between resting memory CD4^+^ T cells and M0 macrophages (**Figure 5**). Combined with prior evidence that CD4^+^ T cells can modulate macrophage polarization toward antitumor phenotypes via IFN-γ secretion (47, 48), we speculate that the activation of resting memory CD4^+^ T cells in the low-risk group may inhibit the M0/M2 transition, thereby reducing the formation of an immunosuppressive microenvironment. This provides new mechanistic support for the coordinated regulation of immune cell populations. In addition, activated mast cells have been reported to release histamine and leukotrienes, which recruit dendritic cells, T cells, and other immune cells to enhance local antitumor immunity (49). The positive correlation between ATP7B and activated mast cells (cor = 0.38), along with the negative correlation between activated mast cells and risk score, suggests that ATP7B may influence the tumor immune microenvironment by modulating mast cell activation.

Importantly, expression levels of nine ICIs differed significantly between risk groups and showed strong correlations with ADME genes and risk scores, providing clinically relevant clues for immunotherapy strategies. Most ICIs, including BCL2 and CD33, were upregulated in the low-risk group, whereas only CHEK1, IDH2, and PLK1 were elevated in the high-risk group. Literature evidence indicates that BCL2, an anti-apoptotic molecule, can reduce T-cell exhaustion and sustain immune responsiveness (50). In contrast, CHEK1 and PLK1 are cell cycle regulators (51, 52), whose high expression is associated with enhanced tumor cell proliferation (52), offering a molecular explanation for the poorer prognosis and potentially reduced responsiveness to conventional immunotherapies in the high-risk group. Furthermore, the finding that most ADME genes were positively correlated with ICIs suggests that these genes may modulate ICI expression or function, thereby influencing the efficacy of immunotherapy.

Collectively, by integrating analyses of immune infiltration, ADME genes, and ICI expression, this study uncovered potential tumor microenvironment–mediated mechanisms driving prognostic disparities in BRCA. These results warrant further validation and may provide both theoretical and experimental foundations for future combination strategies, such as the use of ADME gene modulators in conjunction with ICIs.

To identify potential therapeutic drugs, the variance in IC50 values was calculated across different drugs within the high- and low-risk cohorts, focusing on those with the top 10 *P*-values for differential impact in patients with BRCA (**Figure 6A**). It is worth mentioning that imatinib, which inhibits CYP3A4, has been documented as a combined treatment for individuals with CML and BRCA, without causing additional side effects (53). ABT.263 (navitoclax) synergizes with a novel myeloid cell leukemia sequence 1 (MCL−1) downregulation, significantly inducing intrinsic apoptosis in TNBC cells (54). Future studies on drugs such as AZD.2281, BI.D1870, and IPA.3, are expected to yield improved clinical outcomes in patients with BRCA. A regulatory network encompassing lncRNAs, miRNAs, and mRNAs was constructed and the protein expression of prognostically significant genes was validated (**Figure 6B**). An examination of the expression levels for the six genes associated with prognosis was conducted. The results, displayed in a boxplot format, revealed variations in expression among these genes (**Supplementary Figure S7**). Independent expression analyses at different levels were used to validate our conclusion; qRT-PCR and WB results were consistent with those obtained from TCGA database (**Figure 9**). Specifically, at the level of gene and protein expression, ATP7B showed high expression in BRCA samples, while the other five genes (GSTM2, ADHFE1, ALDH2, NOS1, and ALDH3A1) showed lower expression levels, which was consistent with gene expression results in transcriptome analysis. Interestingly, in a study on drug resistance in BRCA cells also pointed out that the expression level of ATP7B in BRCA tissues was slightly higher than that in normal tissues. In addition, ATP7B is often closely related to adverse reactions such as cisplatin resistance in cancer treatment (55). These results suggest that the prognostic genes verified by experiments have potential value as new therapeutic targets for BRCA and provide a new perspective and idea for the development of BRCA treatment strategies and personalized treatment programs. Moreover, their distinct expression patterns and varying protein contents in BRCA indicated that these six genes had the potential to serve as biomarkers, warranting further investigation. On the other hand, combined with bioinformatics results from public databases and experimental validation, these results suggested that these six genes played different roles in regulating physiological function and prognosis. Although the IHC results were unsatisfactory, fluctuations in protein abundance may be attributed to post-transcriptional mechanisms or additional variables, which may account for discrepancies in gene and protein expression levels (**Figure 10**). Thus, these dates support a deeper exploration of the implicated genes and the intricate controls governing them.

This study utilized bioinformatic techniques to identify ADME-related prognostic genes in BRCA and explored their potential mechanisms of action. Specifically, GSTM2, ADHFE1, ALDH2, NOS1, ATP7B, and ALDH3A1 were linked to BRCA prognosis. Investigating these genes provides new insights and valuable information for BRCA treatment. While our study provides a comprehensive bioinformatic framework and preliminary experimental validation, its immediate clinical translation is limited by the absence of large-scale prospective patient cohorts and functional *in vivo* studies. Therefore, these findings should be interpreted as hypothesis-generating, and further multi-center validation and mechanistic investigations are warranted before clinical implementation. First, prospective clinical validation was insufficient, as no forward-looking validation using breast cancer patient samples was performed, and differences among molecular subtypes were not considered. These factors may partially weaken the model’s translational applicability. Moreover, the clinical sample validation results were not fully consistent with the findings from the WB and IHC assays. Second, drug validation remains in the in-silico stage. Although drug sensitivity prediction was performed based on the GDSC database, no *in vitro* or *in vivo* functional assays were conducted on the candidate compounds. Third, the mechanistic investigation lacked depth, focusing primarily on gene expression confirmation without incorporating functional experiments, such as gene knockdown or drug response assays. In particular, the molecular mechanism underlying the association between elevated ATP7B expression and breast cancer drug resistance has not been explored in detail, thereby limiting the mechanistic persuasiveness of the study. To address these limitations, future studies should involve the prospective collection of clinical samples, considering molecular subtype differences to enhance the model’s clinical applicability. Key predictive compounds will be selected for *in vitro* drug response assays in breast cancer cell lines, and vivo validation will be performed using animal models, if possible. Additionally, with adequate funding support gene knockdown experiments will be designed to investigate the influence of target genes, such as GSTM2 and ATP7B, on the biological behavior of breast cancer cells, thereby improving the functional depth and clinical relevance of the study.

## Data availability statement

The datasets analyzed in this study are available from the UCSC-Xena database (https://xenabrowser.net/datapages/) and GEO database(https://www.ncbi.nlm.nih.gov/geo/).
