# Supplementary material for: Establishment and validation of a prognostic risk model based on ADME-related genes in breast cancer
Source: Front Oncol. 2025 Nov 7;15:1568379. doi: 10.3389/fonc.2025.1568379 (PMC12634386; doi:10.3389/fonc.2025.1568379)
Supplement: Supplementary file 1 [file DataSheet1.docx]

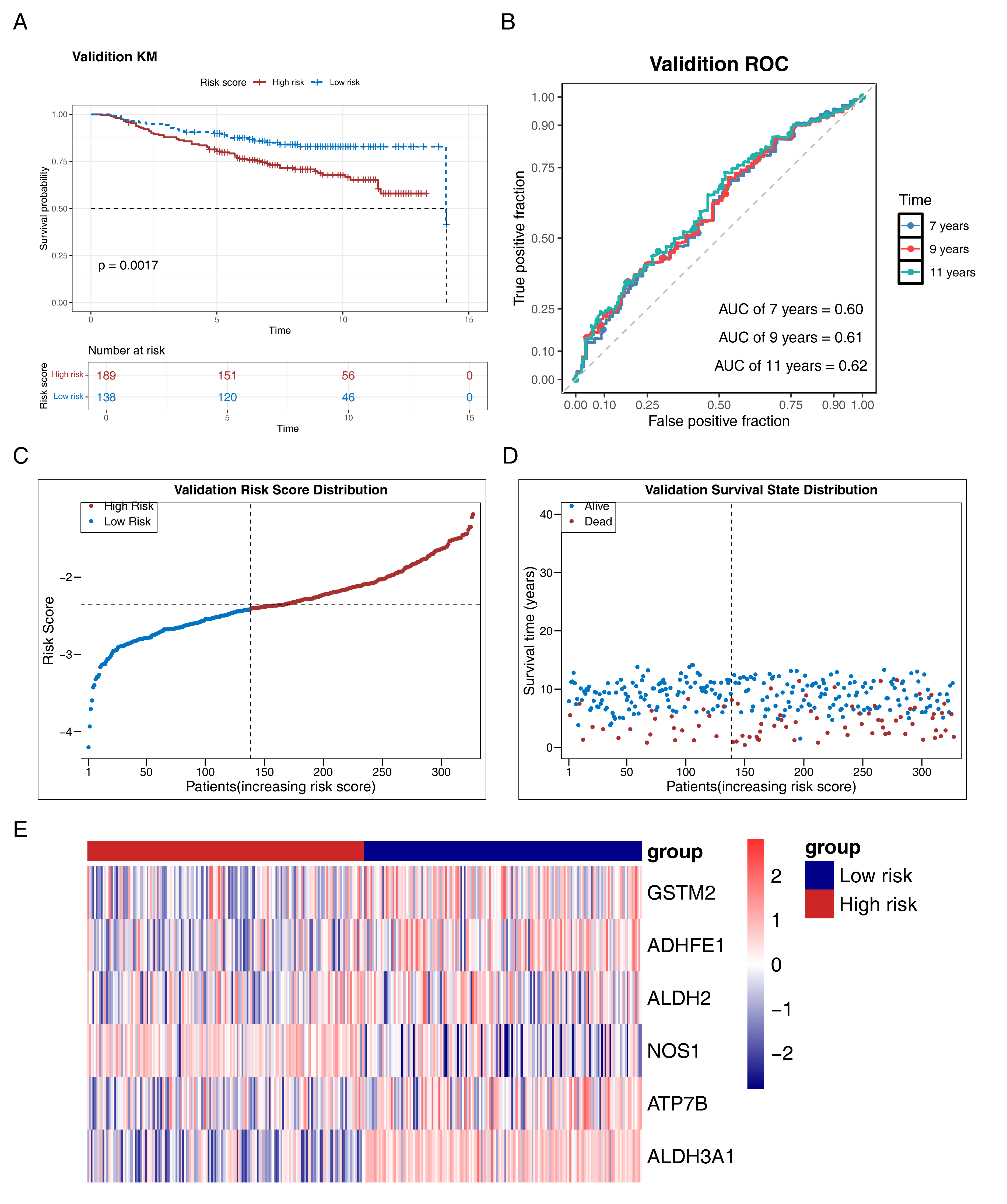


Figure S1: Independent external validation based on the GSE20685 dataset. (A) KM curve. (B) The AUC values confirmed the nomogram's robust clinical predictive capability at 7, 9, and 11 years. (C) Risk profile: Red dots are high-risk samples and blue dots are low-risk samples. (D) Survival status: red dots represent dead samples, and blue dots represent surviving samples. (E) Heatmap of the prognostic gene expression.


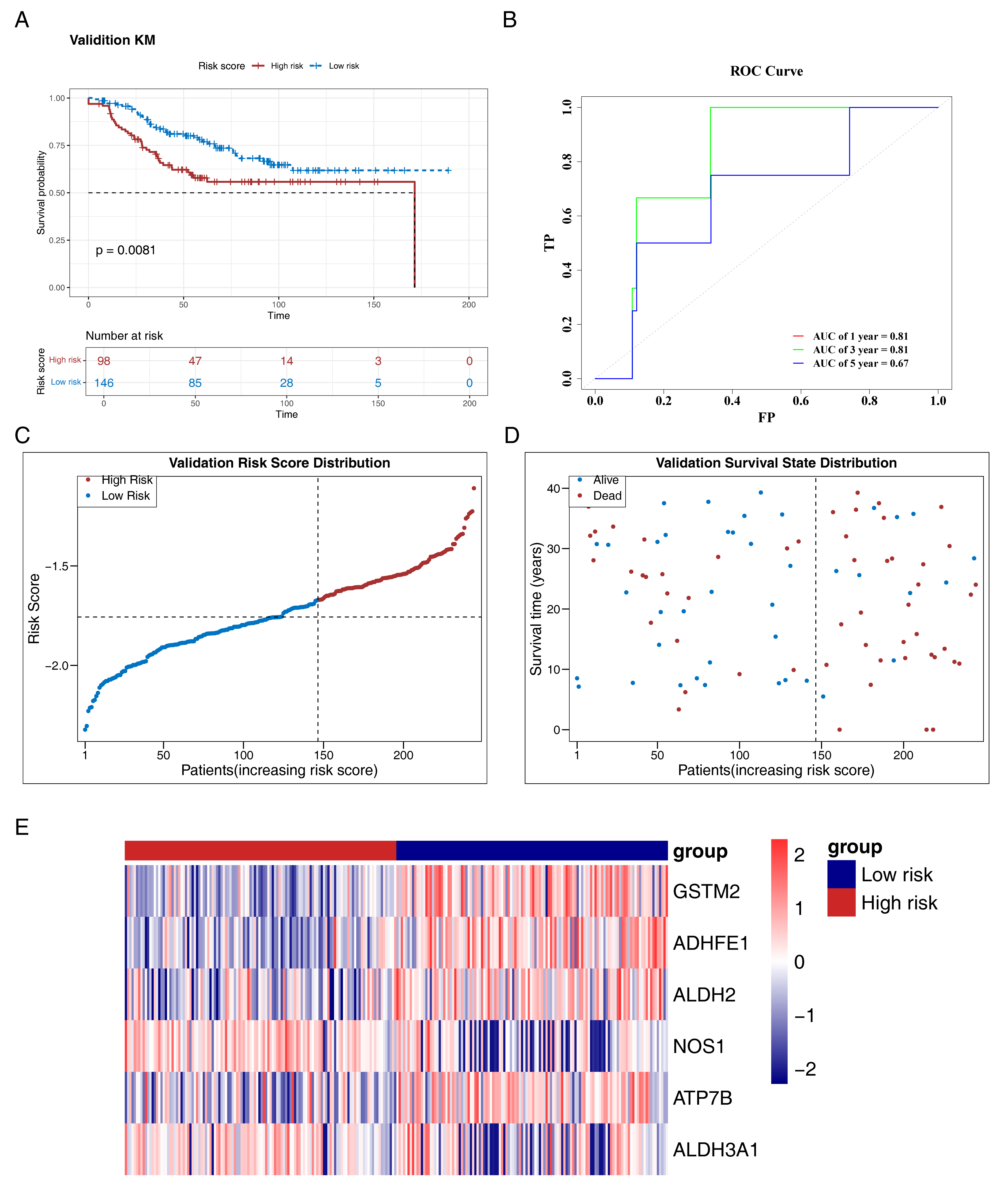


Figure S2: Validation through external assessment utilizing the GSE20685 dataset. (A) Kaplan-Meier plot. (B) AUC measurements verified the nomogram's strong predictive ability in clinical settings at 1, 3, and 5 year intervals. (C) Risk distribution: High-risk cases are indicated by red points, while low-risk cases are shown as blue points. (D) Survival outcomes: Red points signify deceased cases, and blue points represent living cases. (E) Expression heatmap of genes with prognostic significance.


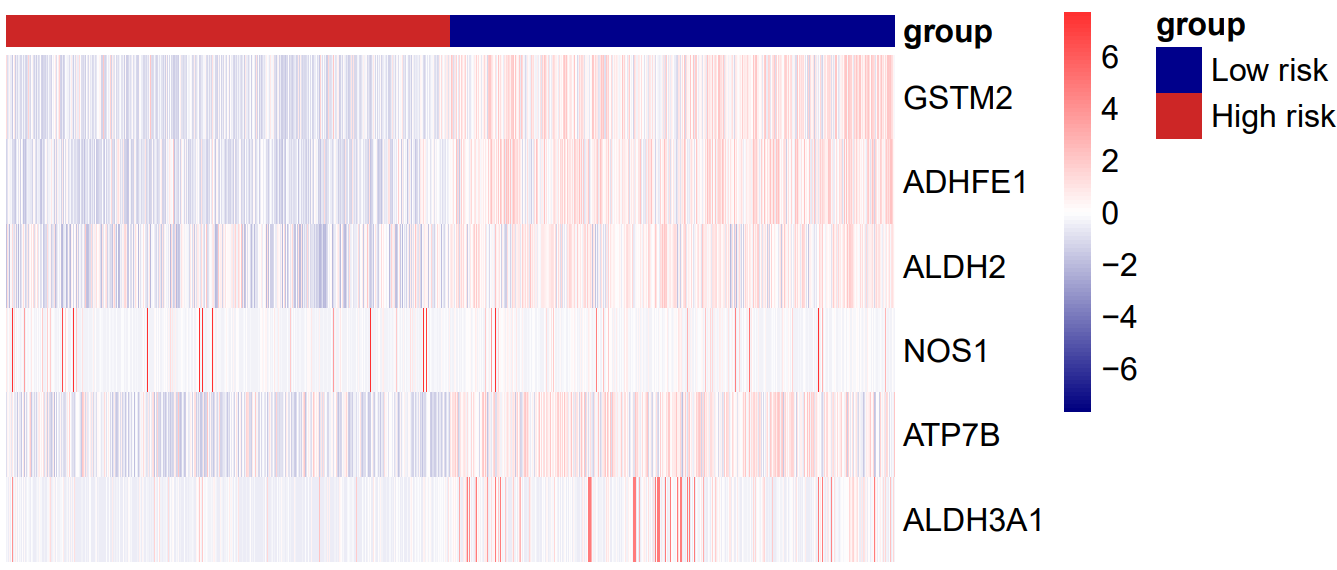


Figure S3: Analysis of prognostic gene expression between the risk groups showed that GSTM2 and ADHFE1 were more highly expressed in the low-risk cohort.


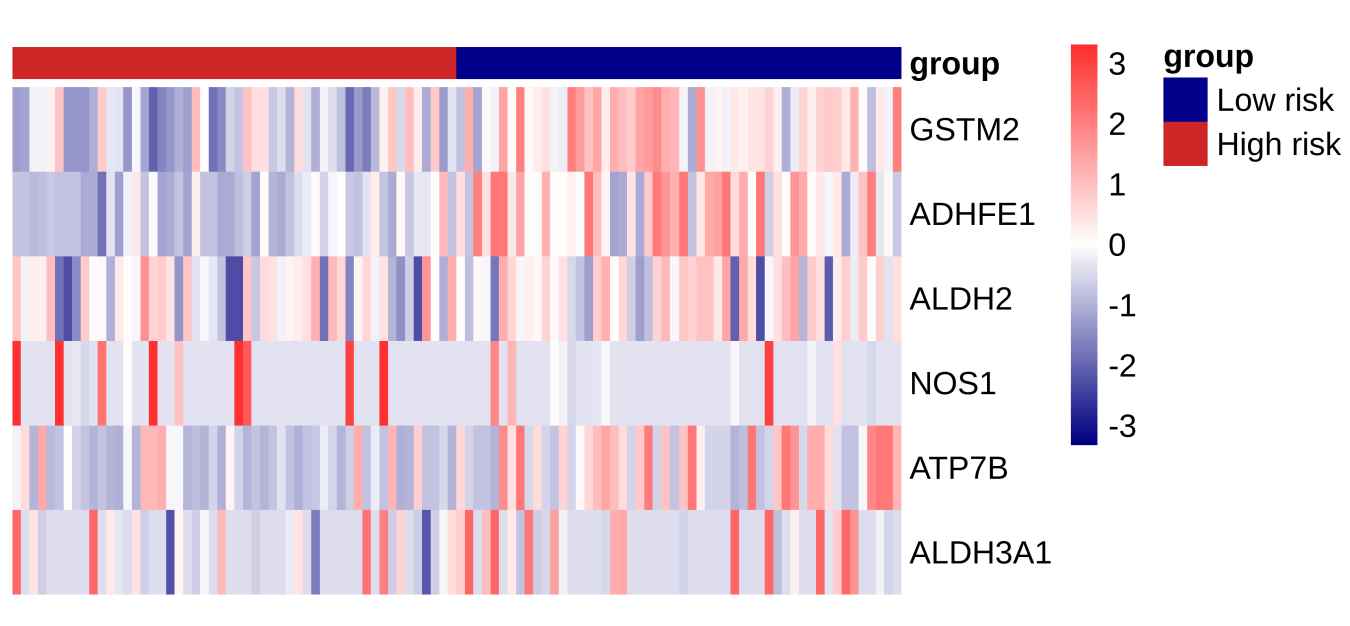


Figure S4: Examination of gene expression patterns associated with prognosis revealed that the low-risk group exhibited higher levels of GSTM2 and ADHFE1 expression compared to the high-risk group.


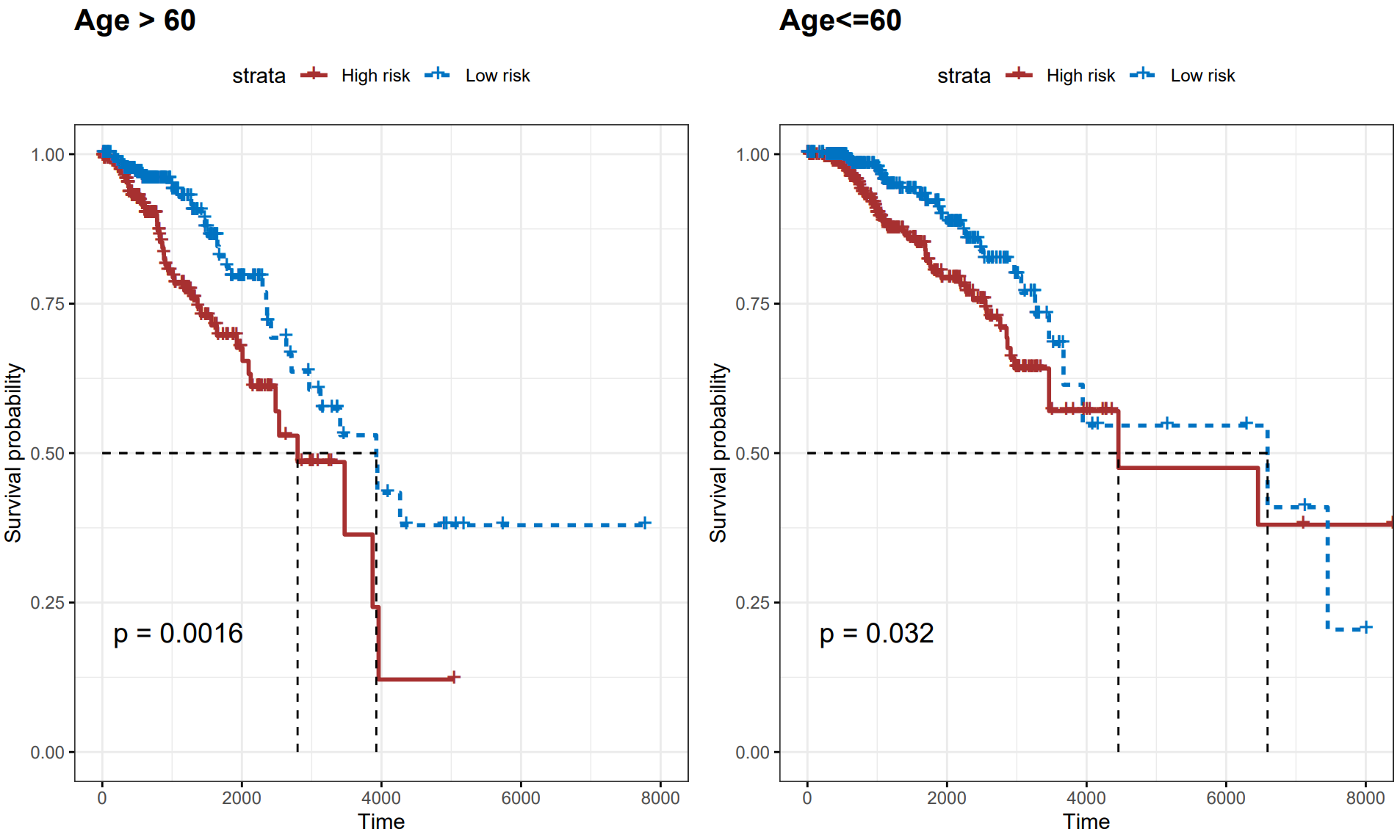


Figure S5: Kaplan-Meier survival analysis, comparing high- and low-risk groups across various clinical factors, revealed that patients in the low-risk category exhibited significantly extended survival rates in both age brackets: those over 60 and those 60 or younger.


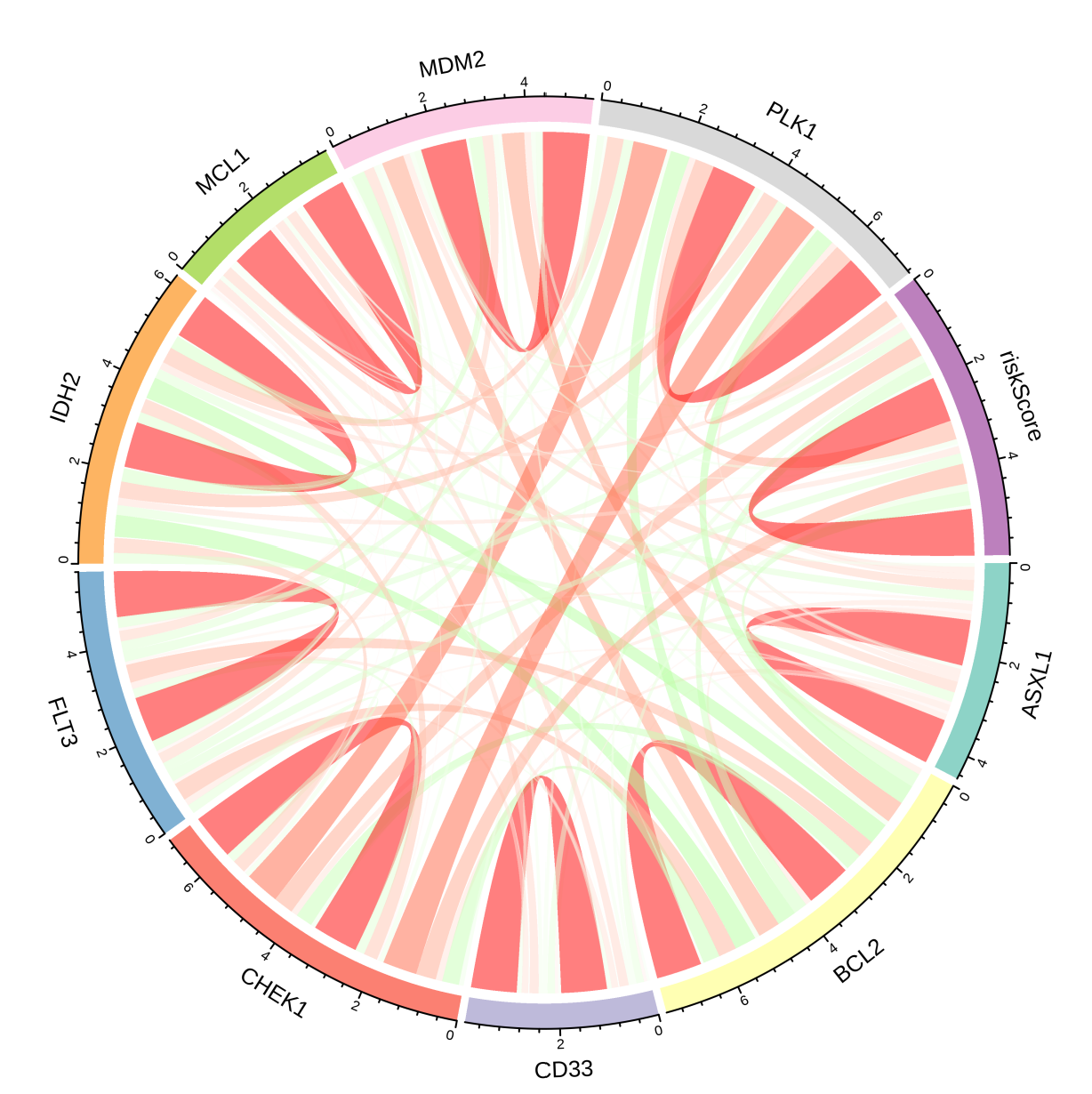


Figure S6: The majority of ADME-related prognostic genes exhibited a positive association with ICIs (correlation coefficient > 0, P < 0.05). However, CHEK1 and PLK1 were exceptions, displaying negative associations (correlation coefficient < 0, P < 0.05).

**qRT-PCR test report**

| **No.** | **Primer** | **Primer sequence (5'-3')** | **Base number** | **Synthetic total (OD)** |
| --- | --- | --- | --- | --- |
| 1 | GSTM2 2946-F | TGTGCGGGGAATCAGAAAAGG | 21 | 2 |
|  | GSTM2 2946-R | CTGGGTCATAGCAGAGTTTGG | 21 | 2 |
| 2 | ADHFE1 137872-F | TGGACTTTCACCTTCTGGGAA | 21 | 2 |
|  | ADHFE1 137872-R | GGAGAGGTTCTTGTCTGTCATCA | 23 | 2 |
| 3 | ALDH2 217, -F | ATGGCAAGCCCTATGTCATCT | 21 | 2 |
|  | ALDH2 217, -R | CCGTGGTACTTATCAGCCCA | 20 | 2 |
| 4 | NOS1 4842, -F | TTCCCTCTCGCCAAAGAGTTT | 21 | 2 |
|  | NOS1 4842, -R | AAGTGCTAGTGGTGTCGATCT | 21 | 2 |
| 5 | ATP7B 540-F | GCCAGCATTGCAGAAGGAAAG | 21 | 2 |
|  | ATP7B 540-R | TGATAAGTGATGACGGCCTCT | 21 | 2 |
| 6 | ALDH3A1 218-F | TGGAACGCCTACTATGAGGAG | 21 | 2 |
|  | ALDH3A1 218-R | GGGCTTGAGGACCACTGAG | 19 | 2 |

Table S1. Primers of the real-time reverse transcription-polymerase chain reaction.

| Case 1: Pathology Number: 2409888, 39 years old, female. The right breast shows grade III invasive carcinoma measuring approximately 4 x 3.5 x 2.3 cm. Additional sections (top, bottom, left, right, and margins) show no evidence of cancer involvement. The sentinel lymph nodes showed negative results (0/3). Note: Immunohistochemical markers (I240524) indicate the following cancer cells: ER (-), PR (-), Her-2 (2+), Ki-67 (+) approximately 90%, E-cadherin (+), P120 membrane (+), and CK5/6 (+). |
| --- |
| Case 2: Pathology Number: 2404616, 43 years old, female. The left breast shows an invasive carcinoma measuring approximately 2.8 cm, non-specific type, grade II-III. The specimen showed tumor invasion at the upper, lower, inner, outer, and chest wall margins, whereas no cancer was observed at the chest wall side margin. Additionally, the sentinel lymph nodes showed positive results (1/3). Note: Immunohistochemical markers (I240195) for the tumor cells are as follows: ER (strong, approximately 90%), PR (moderate intensity, approximately 90%), Her-2 (2), Ki-67 (approximately 30%), E-cadherin (+), P120 (membrane +), CK5/6 (-), P63 (-), P53 (-/+). |
| Case 3: Pathology Number: 2402532, 47 years old, female. The right breast showed an invasive carcinoma of non-specific type, grade II, measuring approximately 4 cm. Note: Immunohistochemical markers (I240125) for the cancer cells are as follows: ER (-), PR (-), Her-2 (2+), Ki-67 (+) approximately 80%, E-cadherin (+), P120 membrane (+), and CK5/6 (-). |
| Case 4: Pathology Number: 2403181, 51 years old, female. The left breast shows invasive carcinoma, grade II-III, non-specific type, measuring 3 x 1.5 x 1 cm, with cancer emboli observed in the blood vessels. Note: Immunohistochemical markers (I240134) for the cancer cells are as follows: ER (+, strong, positive rate approximately 90%), PR (+, moderate intensity, positive rate approximately 90%), Her-2 (2), Ki-67 (+) approximately 5%, E-cadherin (+), P120 membrane (+), and CK5/6 (-). |
| Case 5: Pathology Number: 2404727, 57 years old, female. The left breast shows invasive carcinoma, grade II-III, measuring approximately 1.5 cm, non-specific type, with localized microinvasion. Note: Immunohistochemical markers (I240212) for the cancer cells are as follows: ER (-), PR (-), Her-2 (3), Ki-67 (+) approximately 80%, E-cadherin (+), P120 membrane (+), and CK5/6 (-). |
| Case 6: Pathology Number: 2406357, 29 years old, female. The left breast showed an invasive carcinoma of non-specific type, grade II, measuring approximately 4 cm. Immunohistochemical markers (I240302) for the cancer cells were as follows: ER (0), PR (moderate intensity, +, approximately 10%), Her-2 (2), Ki-67 (approximately 20% +), E-cadherin (+), CK5/6 (+), P63 (-), P53 (wild-type), and 34βE12 (+). |
| Case 7: Pathology Number: 2407550, 51 years old, female. The left breast showed invasive carcinoma, measuring approximately 2 cm, grade II, non-specific type. Note: Immunohistochemical markers (I240357) for the cancer cells are as follows: ER (+, strong, positive rate approximately 90%), PR (+, strong, positive rate approximately 90%), Her-2 (1), Ki-67 (+) approximately 50%, E-cadherin (+), P120 membrane (+), and CK5/6 (-). |
| Case 8: Pathology Number: 2406494, 59 years old, female. The right breast shows an invasive carcinoma, grade II, non-specific type, measuring approximately 1.5 cm. Immunohistochemical markers (I240310) for the cancer cells were as follows: ER (moderate intensity, +, positive rate approximately 80%), PR (moderate intensity, +, positive rate approximately 20%), Her-2 (2+), Ki-67 (+, approximately 60%), E-cadherin (+), P120 (membrane +), and CK5/6 (-). |
| Table S2. Post-operative clinicopathological information for immunohistochemical staining in eight patients. |

**Immunohistochemical test report**

**1. Experimental reagents**

| Reagent | Manufacturer | Article No. |
| --- | --- | --- |
| Anhydrous ethanol | Sinopharm Group | 10009218 |
| Xylene | Sinopharm Group | 10023418 |
| Hematoxylin | Sigma | H9627 |
| Hydrochloric acid | Sinopharm Group | 10011018 |
| Embedded paraffin | Sinopharm Group | 69019361 |
| Neutral gum | Sinopharm Group | 10004160 |
| Immunochromogenic reagent iVision™ Poly-HRP Sheep anti-rat/rabbit secondary antibody reagents | Psychic creature | DD13 |
| DAB color development kit | Servicebio | G1212-200T |
| GSTM2 | Bioss | Bs-16340R |
| ADHFE1 | Wuhan Sanying Biotechnology Co., Ltd. | 14674-1-AP |
| ALDH2 | Wuhan Sanying Biotechnology Co., Ltd. | 15310-1-AP |
| NOS1 | Wuhan Sanying Biotechnology Co., Ltd. | 29231-1-AP |
| ATP7B | Bioss | Bs-1718R |
| ALDH3A1 | Wuhan Sanying Biotechnology Co., Ltd. | 15578-1-AP |

**2.**

| GSTM2 | 1:200 |
| --- | --- |
| ADHFE1 | 1:200 |
| ALDH2 | 1:200 |
| NOS1 | 1:500 |
| ATP7B | 1:200 |
| ALDH3A1 | 1:200 |

Table S3. Corresponding antibody dilution ratio.


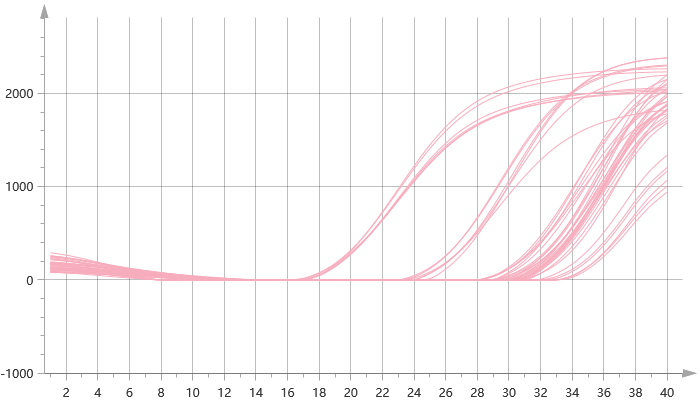


Table S4.MCF 7 amplification curve.


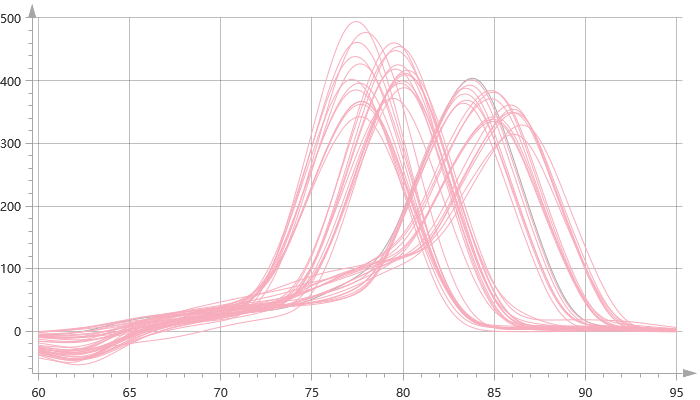


Table S5. MCF 7 dissolution curve.


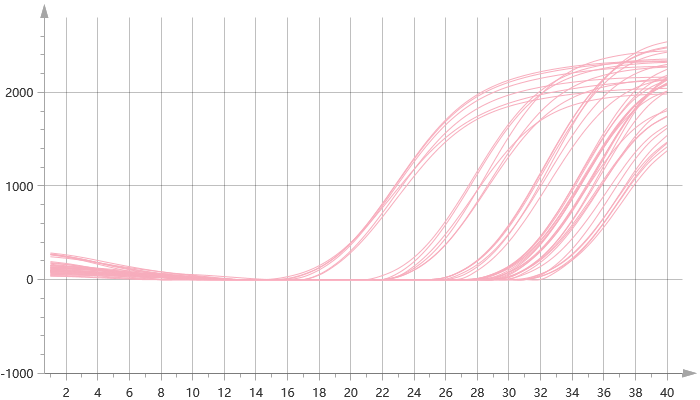


Table S6. T47D amplification curve.


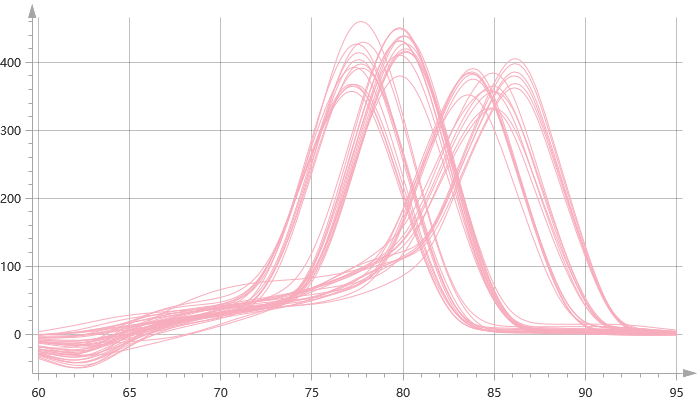


Table S7. T47D dissolution curve.

**The results of western bolt for six proteins.**


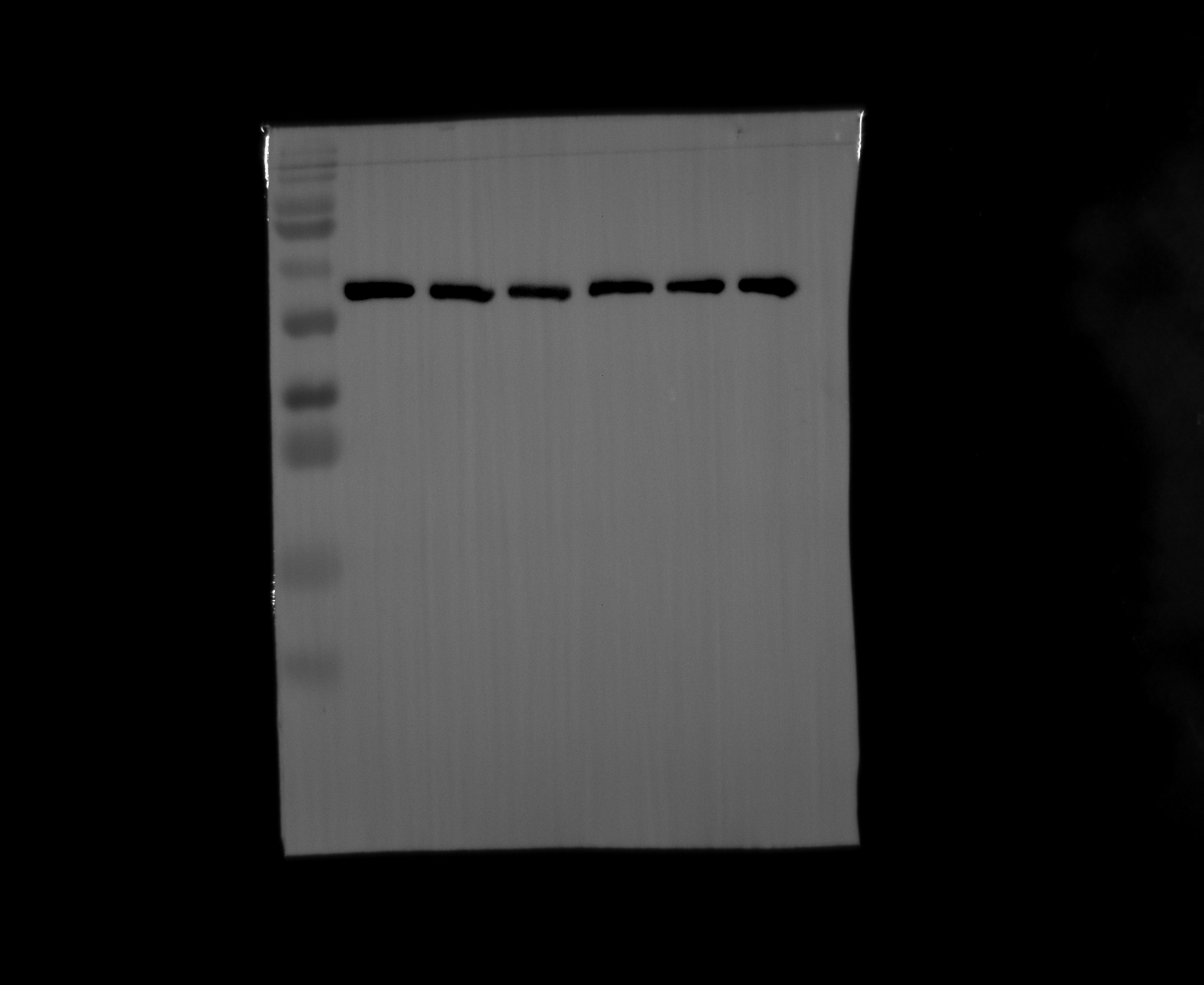


Figure S7: β-actin-1


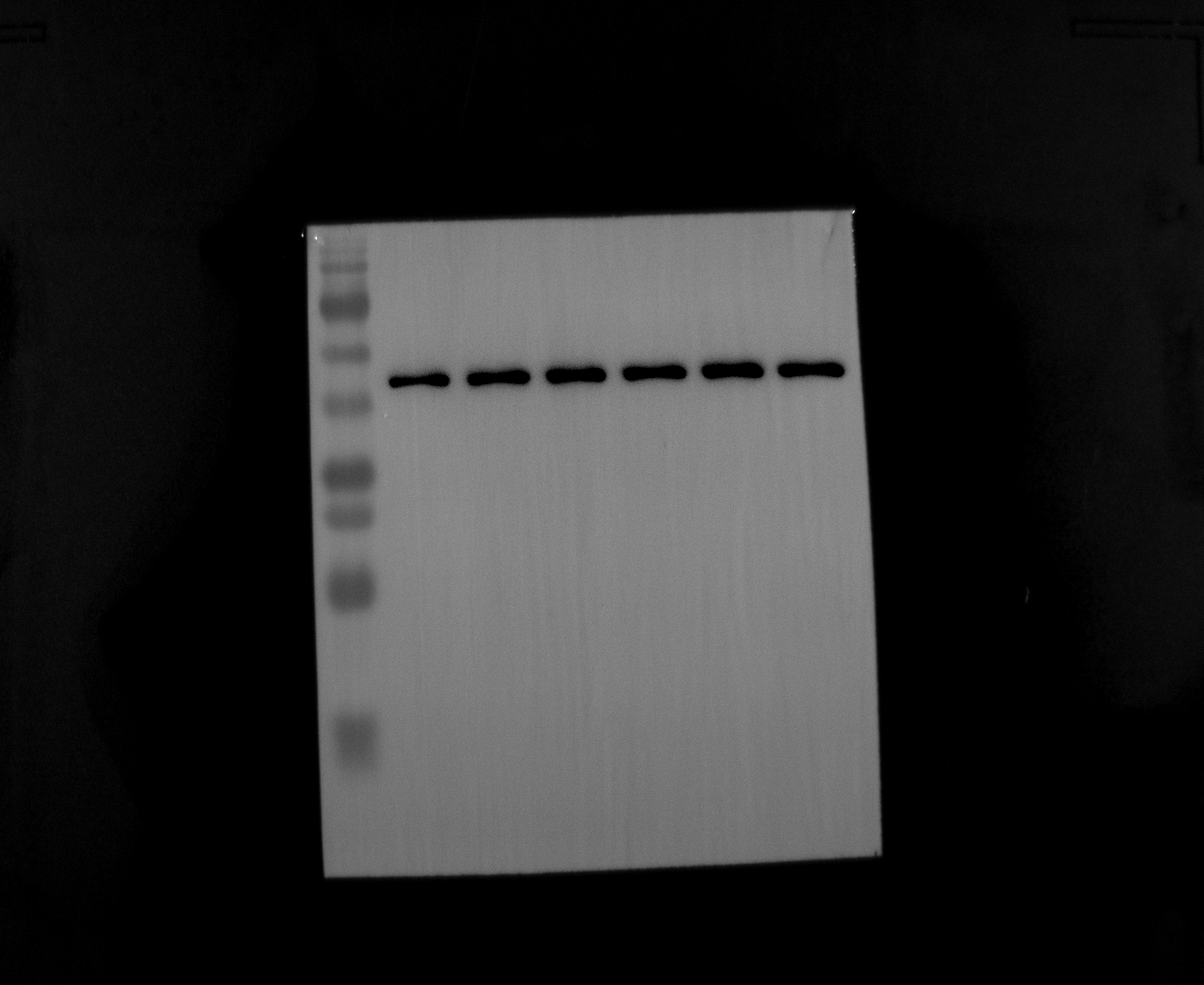


Figure S8: β-actin-2


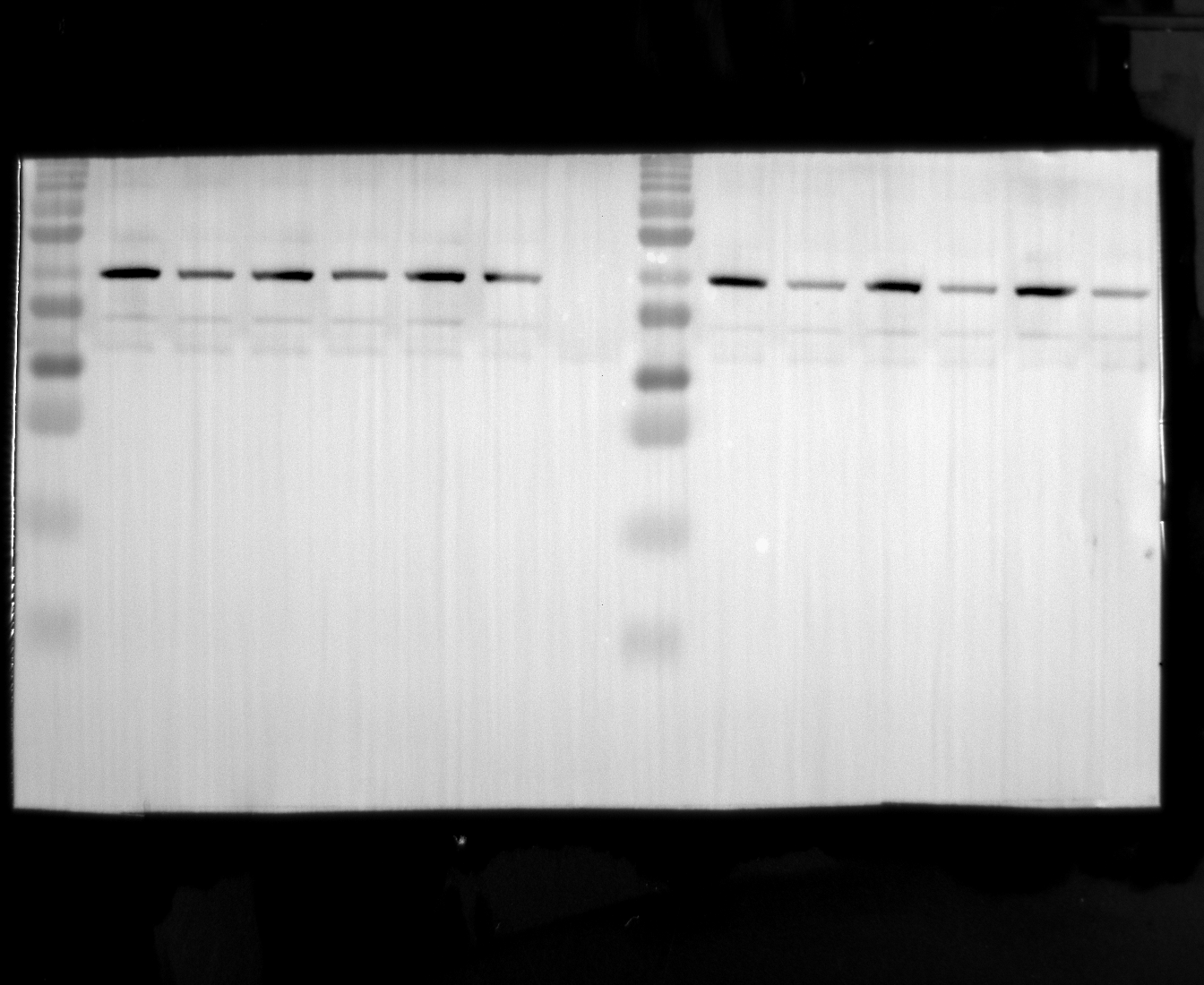


Figure S8: ADHFE1（1-2）


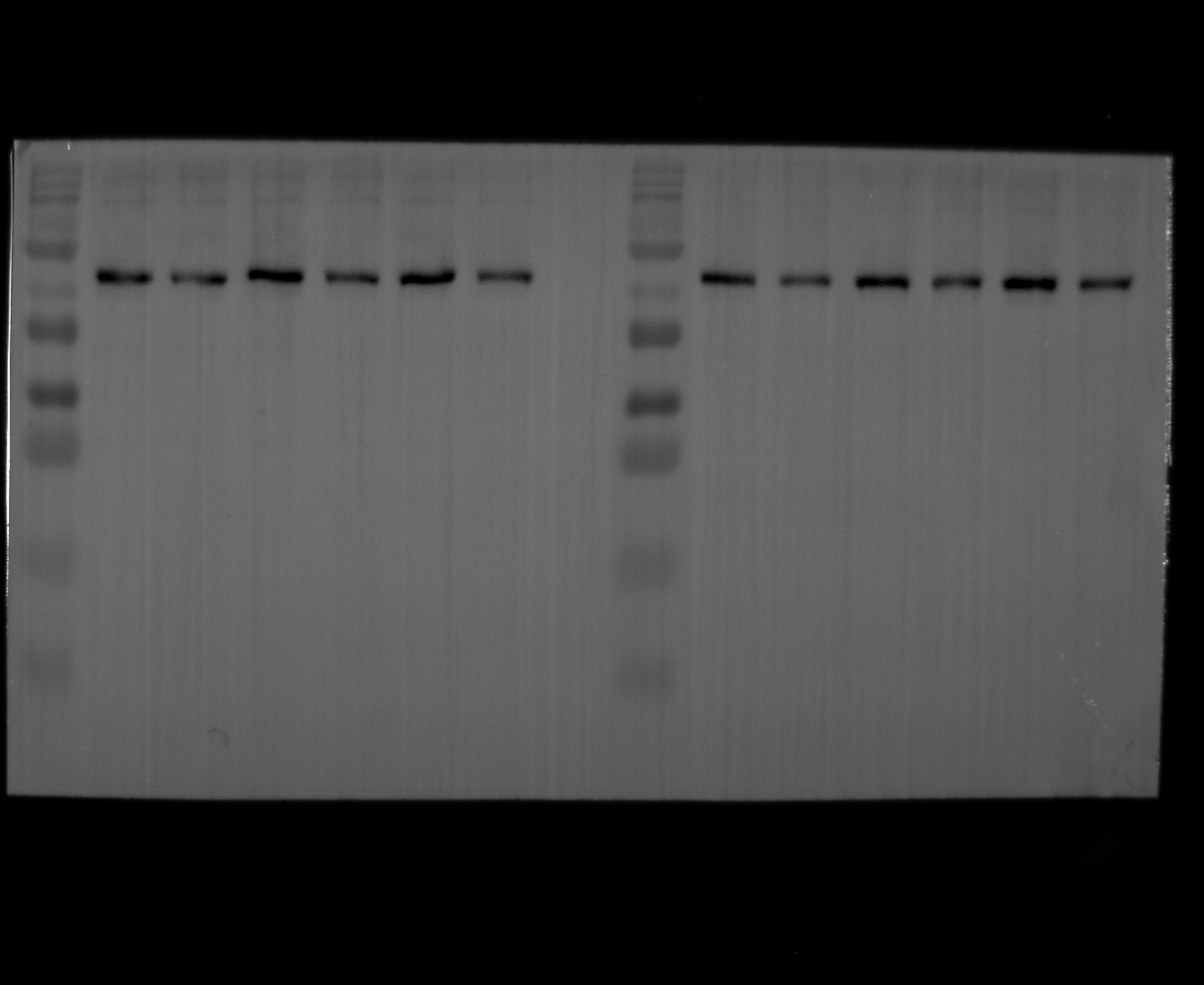


Figure S9: ALDH2（1-2）


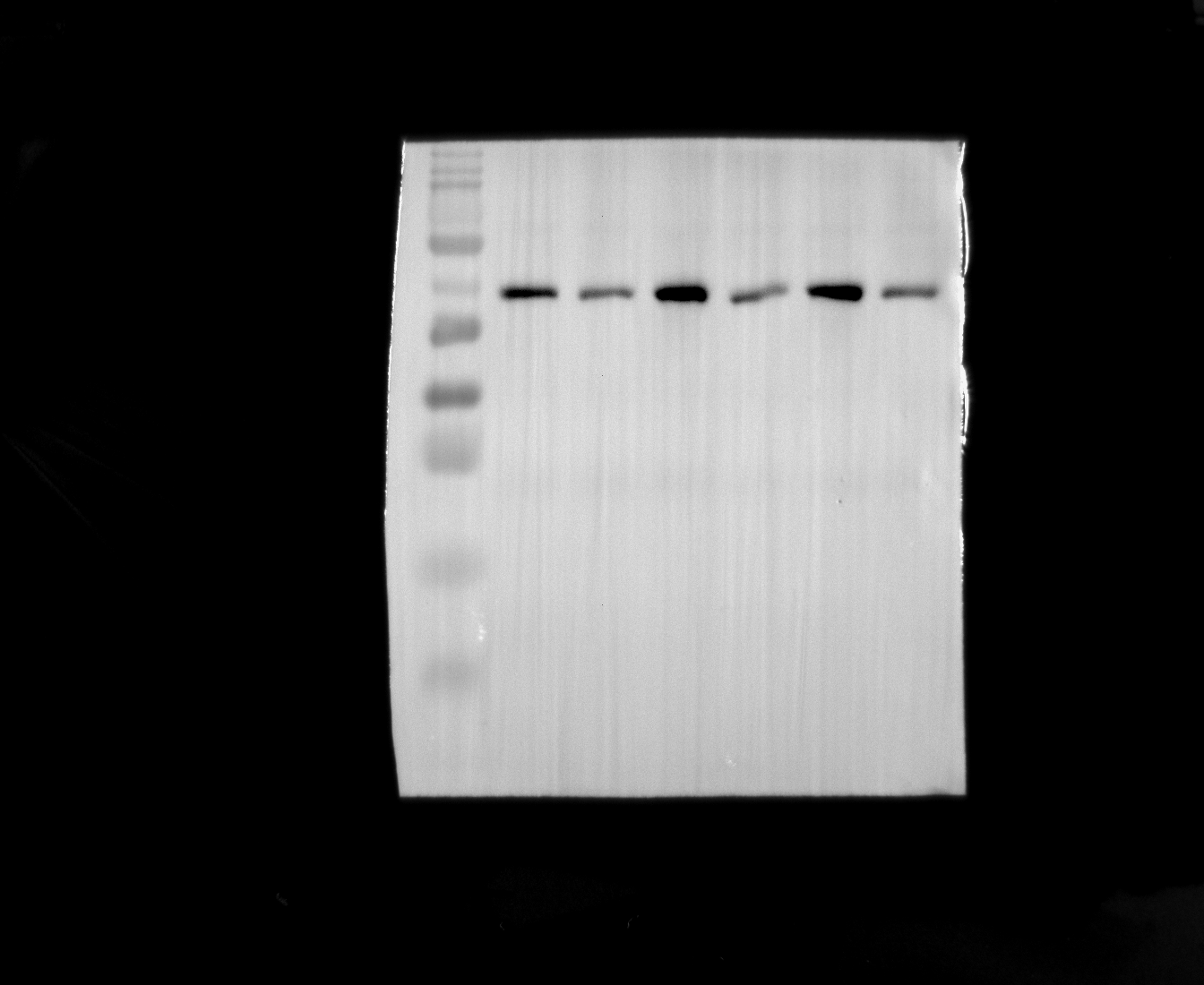


Figure S10: ALDH3A1-1


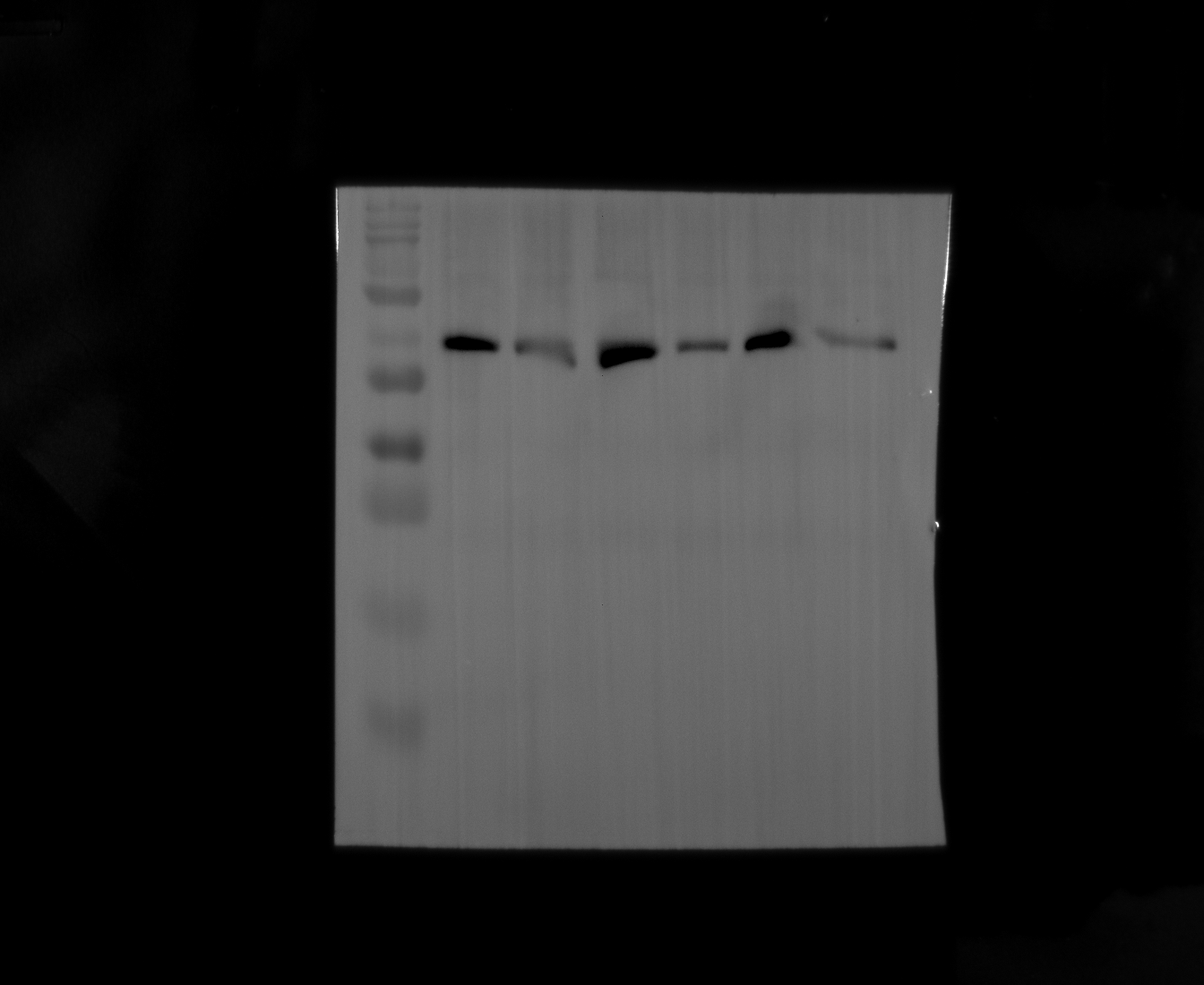


Figure S10: ALDH3A1-2


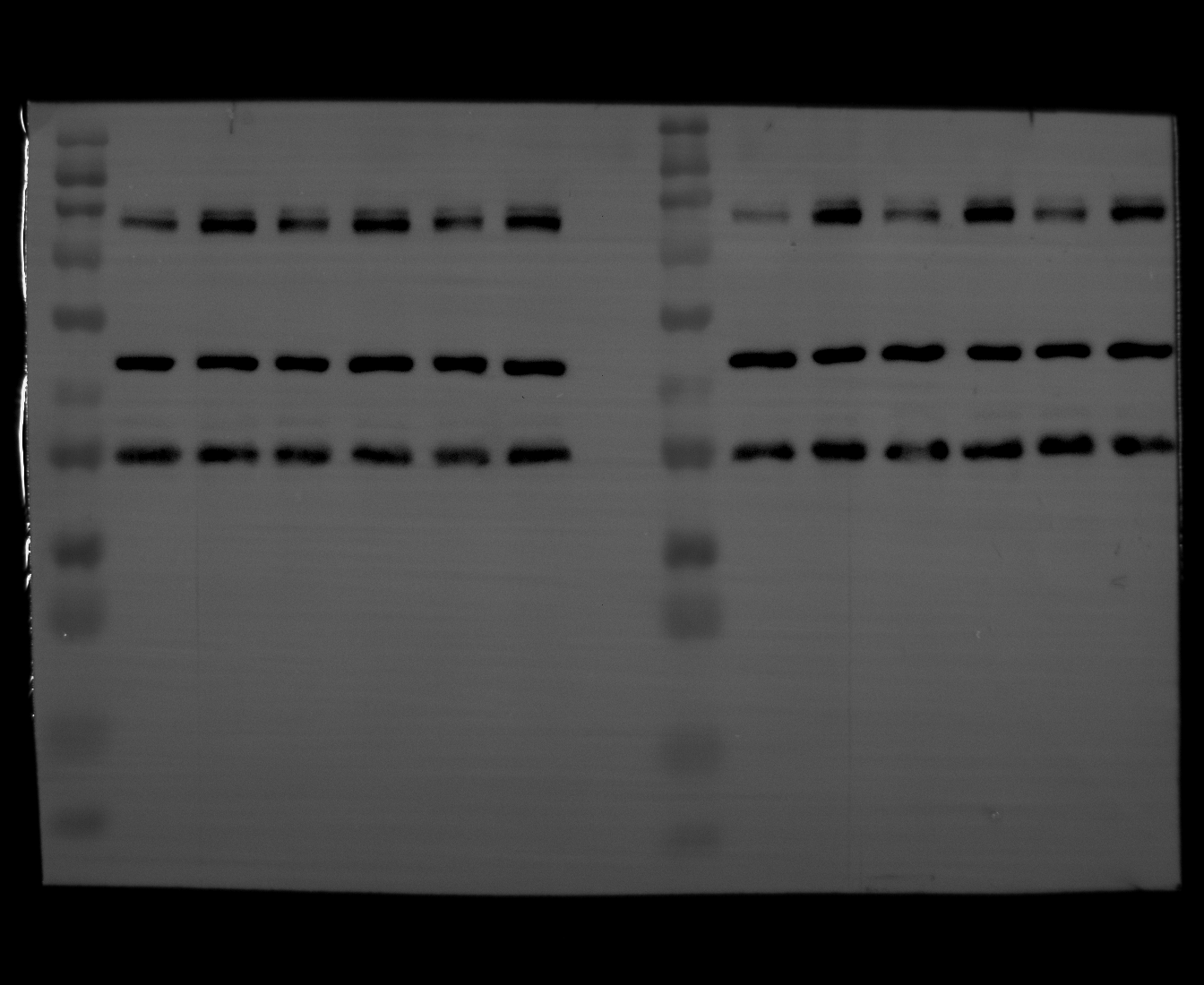


Figure S11: ATP7B（1-2）


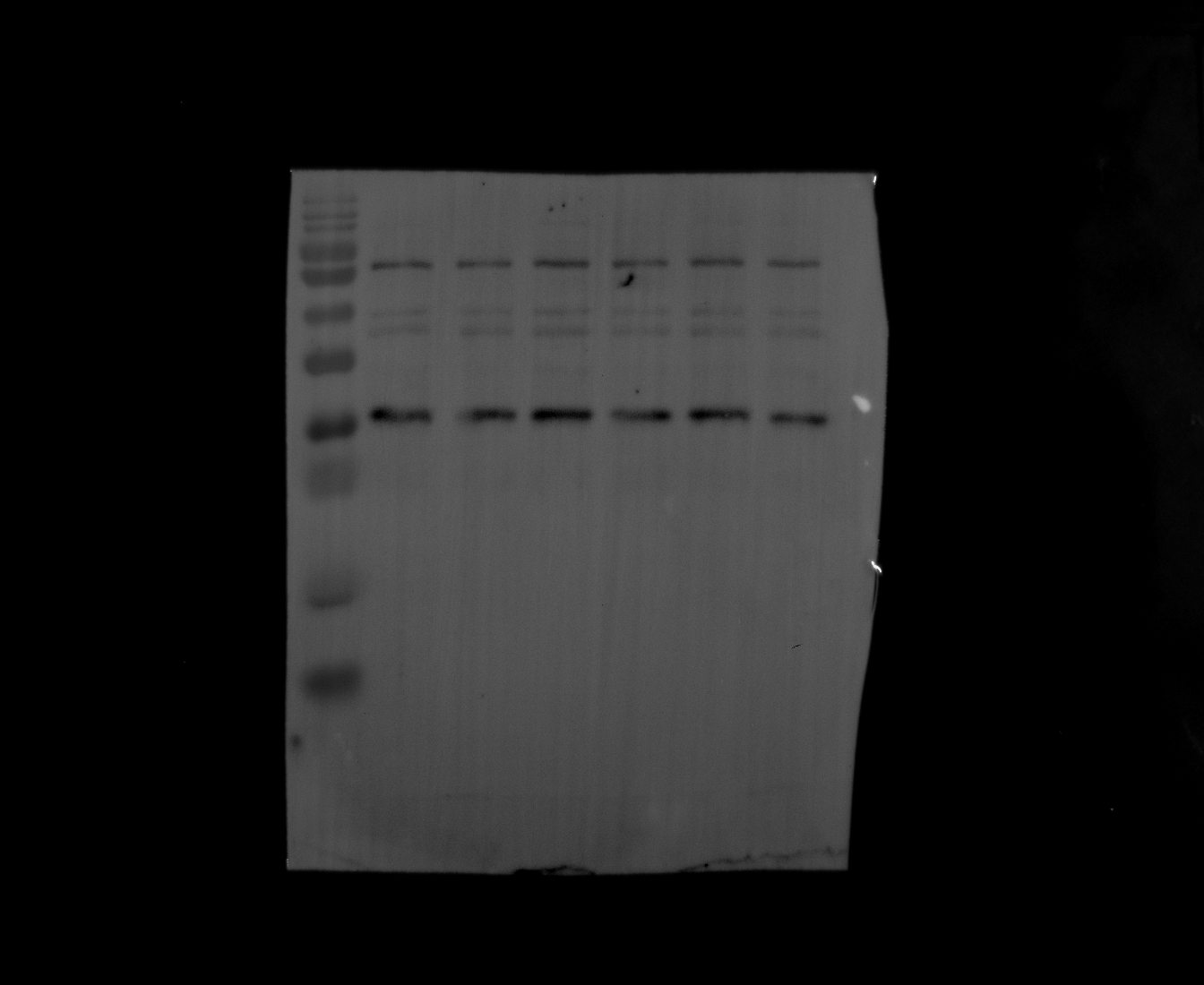


Figure S12: GSTM2-1


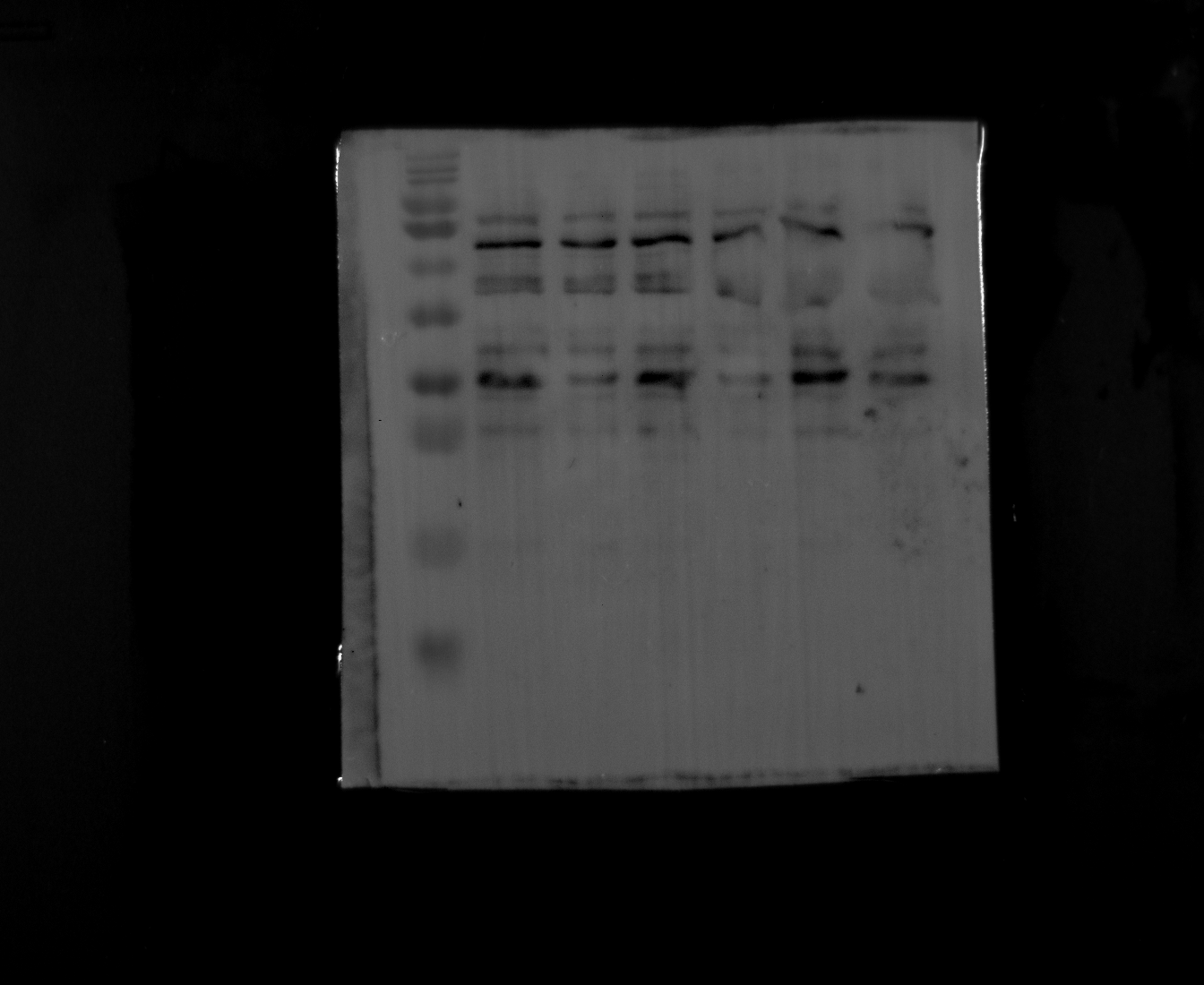


Figure S12: GSTM2-2


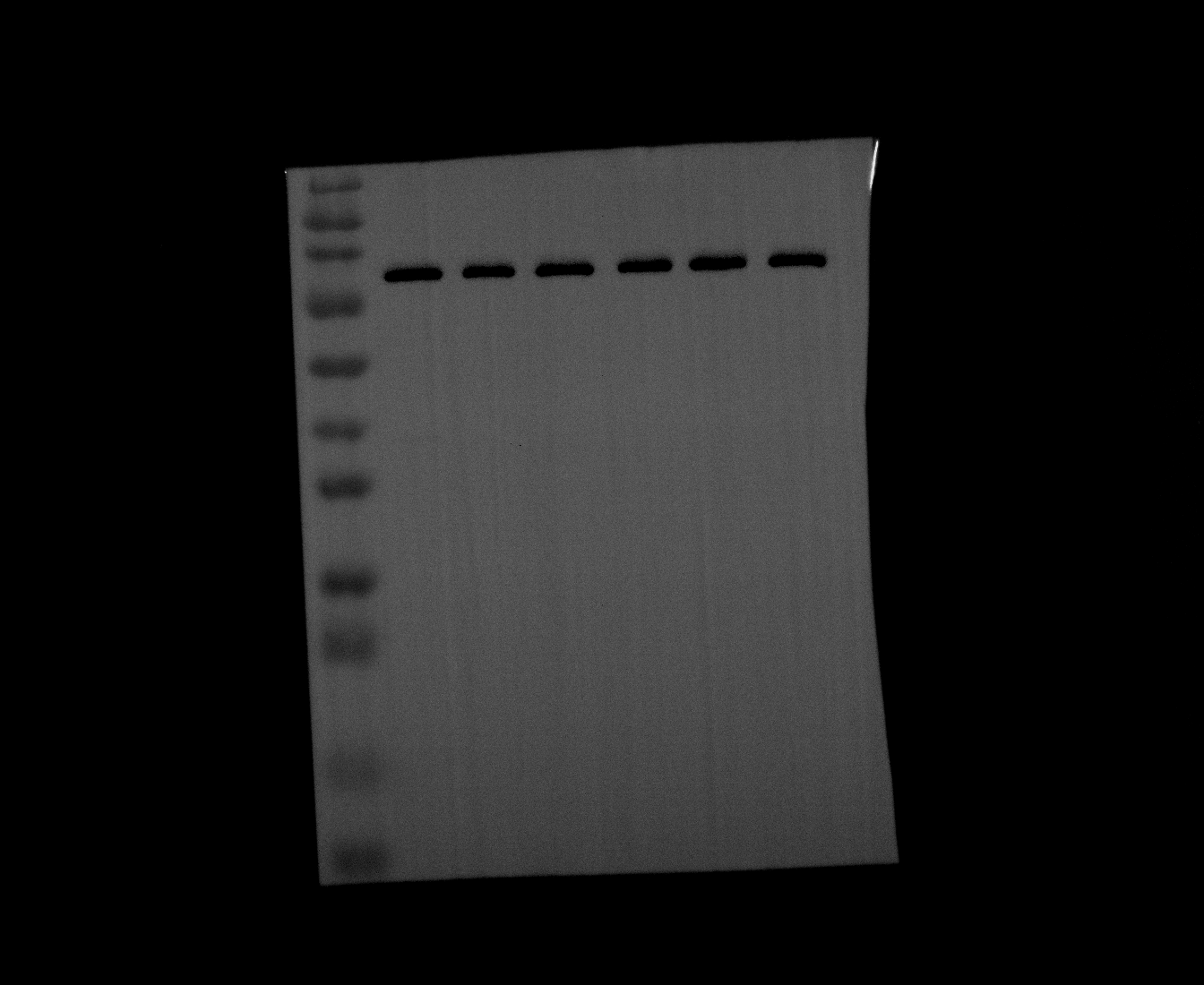


Figure S13: NOS1-1


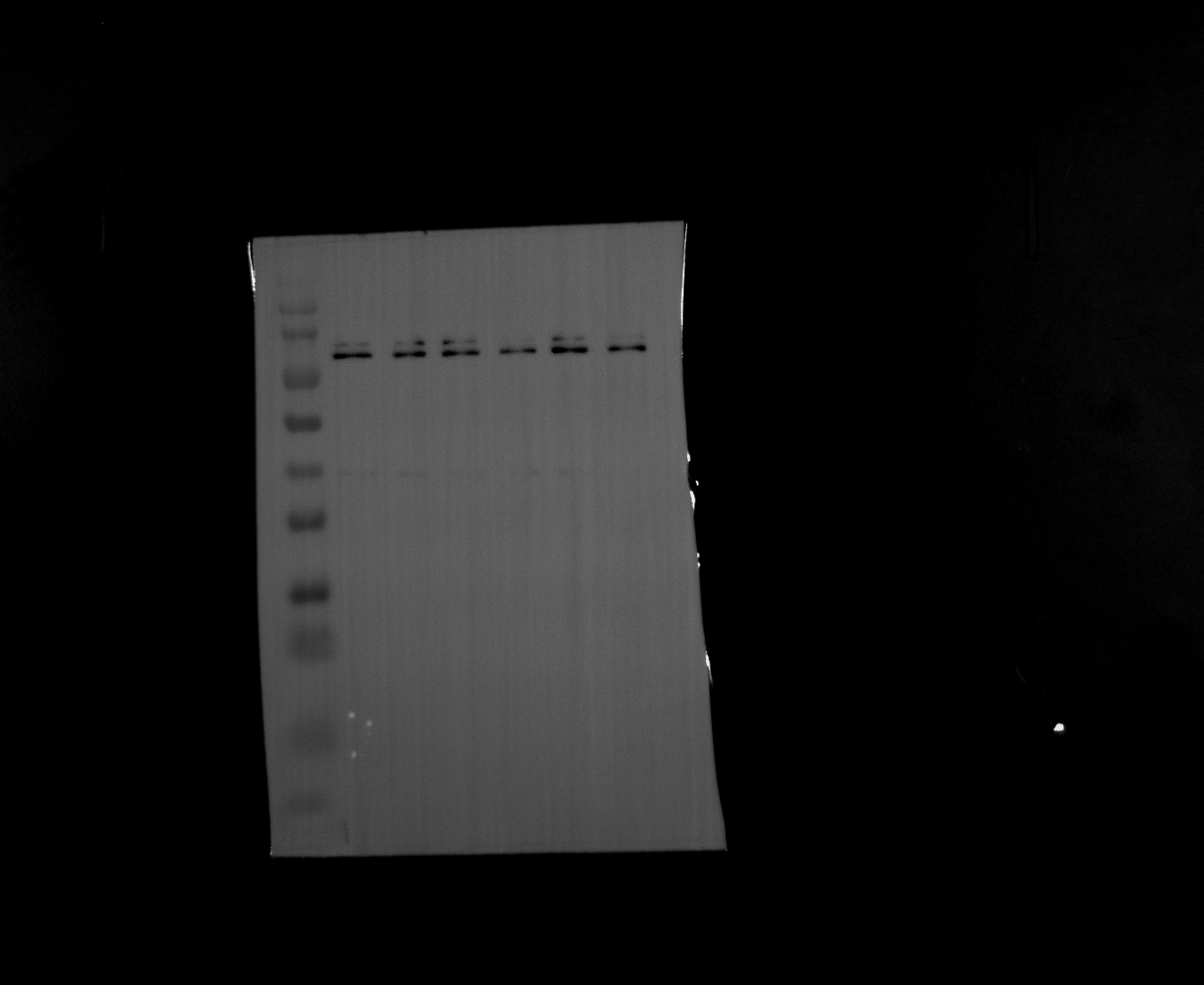


Figure S14: NOS1-2
